# Supplementary material for: Evaluating N‐difluoromethyltriazolium triflate as a precursor for the synthesis of high molar activity [18F]fluoroform
Source: J Labelled Comp Radiopharm. 2021 Sep 20;64(12):466–76. doi: 10.1002/jlcr.3939 (PMC9293032; doi:10.1002/jlcr.3939)
Supplement: Supplementary file 1 — Table S1 Radiochemical yield (%) of dry [18F]fluoride depending on solvent and base/complexant amounts; a trapping volume = 900‐x μL organic solvent + x μL base cryptand stock solution (see main manuscript); b At amounts of 0.015 ‐ 0.15 μmol K2CO3 and 0.035‐0.35 μmol K222 in 900μL MeCN the trapping efficiency was not influenced by the amount of base cryptand complex. Table S2 Radiochemical yield depending on the reaction temperature; 10 min, 0.15 μmol K2CO3, 0.35 μmol K222, 1mM triazolium precursor, 1 mL MeCN (0.1% water); n = 3. Table S3 Radiochemical yield depending on the precursor concentration; reaction conditions: 10 min, 0.15 μmol K2CO3, 0.35 μmol K222, 40°C, 1 mL MeCN (0.1% water); n = 3 (n = 6 for entry 4 and 6). Table S4 Radiochemical yield and molar activity depending on the amount of base/complexant; reaction conditions: 10 min, 40°C, 1mM triazolium precursor, 1 mL MeCN (0.1% water); n = 3. Table S5 Molar activity and radiochemical yield of [18F]fluoroform synthesised from 5 GBq [18F]fluoride under different conditions; 990 μL solvent, 10 μL base complexant stock solution, 1mM triazolium precursor; n = 3 Table S6 Molar activity and radiochemical yield of fluoroform synthesised from 25 GBq [18F]fluoride under different conditions, t = 10 min; * Volume was determined as follows: V (solvent) = 1 mL ‐ (volume of base cryptand stock solution) ‐ (100 μL triazolium solution). Figure S1 1H NMR spectrum of the reaction mixture (Table 1, entry 1, main manuscript). Figure S2 Vertical expansion of Figure S1 (Table 1, entry 1, main manuscript). Figure S3 1H NMR spectrum of the reaction mixture (Table 1, entry 2, main manuscript). Figure S4 Vertical expansion of Figure S3 (Table 1, entry 2, main manuscript). Figure S5 1H NMR spectrum of the reaction mixture (Table 1, entry 3, main manuscript). Figure S6 Vertical expansion of Figure S5 (Table 1, entry 3, main manuscript). Figure S7 1H NMR spectrum of the reaction mixture (Table 1, entry 4, main manuscript). Figure S8 V [file JLCR-64-466-s002.docx]

Supporting Information for:

Evaluating *N*-difluoromethyltriazolium triflate as a precursor for the synthesis of high molar activity [^18^F]fluoroform

Anna Pees^1^, Maria J.W.D. Vosjan^2^, Jin Young Chai^3^, Hyojin Cha^3^, Dae Yoon Chi^3^, Albert D. Windhorst^1^ and Danielle J. Vugts^1^

*^1^ Amsterdam UMC, VU University, Radiology and Nuclear Medicine, Radionuclide Center, De Boelelaan 1085c, Amsterdam, The Netherlands.*

*^2^ BV Cyclotron VU, De Boelelaan 1081,1081 HV Amsterdam, The Netherlands.*

*^3^ Department of Chemistry, Sogang University, 35 Baekbeomro Mapogu, Seoul 04107, Korea.*

**Table of Contents**

[1 Radiochemistry optimisation 2](#_Toc73516127)

[1.1 Drying of [^18^F]fluoride via the [^18^F]triflyl fluoride method 2](#_Toc73516128)

[1.2 Optimisation of reaction temperature using 500 MBq [^18^F]fluoride 2](#_Toc73516129)

[1.3 Optimisation of precursor concentration using 500 MBq [^18^F]fluoride 3](#_Toc73516130)

[1.4 Optimisation of base and complexant amount using 5 GBq [^18^F]fluoride 3](#_Toc73516131)

[1.5 Optimisation of other parameters using 5 GBq [^18^F]fluoride 4](#_Toc73516132)

[1.6 Optimisation of various parameters using 25 GBq [^18^F]fluoride 5](#_Toc73516133)

[1.7 Calculations concerning base consumption during [^18^F]fluoride release from [^18^F]triflyl fluoride 6](#_Toc73516134)

[2 ^1^H NMR experiments 7](#_Toc73516135)

[2.1 Stability of triazolium precursor 4 in presence of K_2_CO_3_ and K_222_ 7](#_Toc73516136)

[2.2 ^1^H NMR experiments for determining the reaction order 11](#_Toc73516137)

[3 HPLC chromatograms 12](#_Toc73516138)

[3.1 1-(Difluoromethyl)-3-methyl-4-phenyl-1*H*-1,2,3-triazol-3-ium triflate 12](#_Toc73516139)

[3.2 α-[^18^F](Trifluoromethyl)benzhydrol 12](#_Toc73516140)

[3.3 Reaction mixture 13](#_Toc73516141)

# Radiochemistry optimisation

## Drying of [^18^F]fluoride via the [^18^F]triflyl fluoride method

Dry [^18^F]fluoride was produced as described in the experimental section of the main manuscript. Table S1 gives an overview of the radiochemical yields (RCY) of dry [^18^F]fluoride depending on the solvent and the amounts of base and cryptand used in the trapping solution.

Table S1 Radiochemical yield (%) of dry [^18^F]fluoride depending on solvent and base/complexant amounts; ^a^ trapping volume = 900-x µL organic solvent + x µL base cryptand stock solution (see main manuscript); ^b^ At amounts of 0.015 - 0.15 µmol K_2_CO_3_ and 0.035 - 0.35 µmol K_222_ in 900 µL MeCN the trapping efficiency was not influenced by the amount of base cryptand complex.

| Entry | Solvent*^a^* | Trapping temp.  (°C) | Base  (µmol) | Complexant  (µmol) | RCY±SD  (%, dc) | n |
| --- | --- | --- | --- | --- | --- | --- |
| 1 | MeCN | -40 | K_2_CO_3_,  0.015 - 0.15*^b^* | K_222_,  0.035 - 0.35 *^b^* | 69±2 | 15 |
| 2 | THF | -100 | K_2_CO_3_, 0.15 | K_222_, 0.35 | 94±3 | 3 |
| 3 | DMF | -60 | K_2_CO_3_, 0.15 | K_222_, 0.35 | 53±4 | 3 |
| 4 | MeCN | -40 | KHCO_3_, 0.3 | K_222_, 0.35 | 67±1 | 3 |
| 5 | MeCN | -40 | K_2_CO_3_, 0.15 | 18-cr-6, 0.35 | 67±3 | 3 |
| 6 | MeCN | -40 | K_2_CO_3_, 0.75 | K_222_, 1.75 | 73±1 | 3 |
| 7 | MeCN | -40 | K_2_CO_3_, 1.5 | K_222_, 3.5 | 76±8 | 3 |

## Optimisation of reaction temperature using 500 MBq [^18^F]fluoride

[^18^F]Triflyl fluoride was obtained as described in the main manuscript and trapped in 1-1.5 mL MeCN at -40 °C. From this solution, 100 µL were transferred to a vessel containing 890 µL MeCN and 10 µL K_2_CO_3_/K_222_ stock solution (0.15 µmol K_2_CO_3_ and 0.35 µmol K_222_; see main manuscript). The vessel was heated to the required temperature (-20 °C to 100 °C) and 100 µL 10 mM triazolium precursor **4** in MeCN was added. After 10 minutes reaction time [^18^F]fluoroform was purged out of the solution with a helium flow of 10 mL/min. It was led over a silica plus long cartridge and was trapped in 1 mL DMF cooled to -60 °C.

Table S2 Radiochemical yield depending on the reaction temperature; 10 min, 0.15 µmol K_2_CO_3_, 0.35 µmol K_222_, 1 mM triazolium precursor, 1 mL MeCN (0.1% water); n=3.

| Entry | Temp.  °C | RCY±SD  % (dc) |
| --- | --- | --- |
| 1 | -20 | 1±0 |
| 2 | 0 | 10±1 |
| 3 | 20 | 42±15 |
| 4 | 40 | 52±6 |
| 5 | 60 | 45±5 |
| 6 | 80 | 28±13 |
| 7 | 100 | 18±16 |

## Optimisation of precursor concentration using 500 MBq [^18^F]fluoride

[^18^F]Triflyl fluoride was obtained as described in in the main manuscript and was trapped in 1-1.5 mL MeCN at -40 °C. From this solution, 100 µL were transferred to a vessel containing 690-870 µL MeCN and 10 µL K_2_CO_3_/K_222_ stock solution (0.15 µmol K_2_CO_3_ and 0.35 µmol K_222_; see main manuscript). The vessel was heated to 40 °C and 20-200 µL 10 mM or 50-100 µL 100 mM triazolium precursor **4** in MeCN was added. After 10 minutes reaction time [^18^F]fluoroform was purged out of the solution with a helium flow of 10 mL/min. It was led over a silica plus long cartridge and was trapped in 1 mL DMF cooled to -60 °C. The radiochemical yield was determined by dividing the decay-corrected radioactivity of the trapped [^18^F]fluoroform in DMF by the radioactivity of reaction solution before distillation.

Table S3 Radiochemical yield depending on the precursor concentration; reaction conditions: 10 min, 0.15 µmol K_2_CO_3_, 0.35 µmol K_222_, 40 °C, 1 mL MeCN (0.1% water); n = 3 (n = 6 for entry 4 and 6).

| Entry | Prec. conc.  mM | RCY±SD  % (dc) |
| --- | --- | --- |
| 1 | 0.2 | 34±5 |
| 2 | 0.5 | 37±5 |
| 3 | 0.75 | 44±5 |
| 4 | 1 | 45±4 |
| 5 | 2 | 45±3 |
| 6 | 5 | 17±14 |
| 7 | 10 | 2±0 |

## Optimisation of base and complexant amount using 5 GBq [^18^F]fluoride

Dry [^18^F]fluoride was obtained as described in in the main manuscript by trapping [^18^F]triflyl fluoride in 900-**x** µL MeCN and **x** µL K_2_CO_3_/K_222_ stock solution (see main manuscript). The trapping vial was heated to 40 °C and 100 µL 10 mM triazolium precursor **4** in MeCN was added. After 10 minutes reaction time [^18^F]fluoroform was purged out of the solution with a helium flow of 10 mL/min. It was led over a silica plus long cartridge and an ascarite column and was trapped at -60 °C in a vessel containing 900 µL DMF and 2 mg (10 µmol) benzophenone. To the vessel was then added 100 µL of 0.3 M potassium *tert*-butoxide in DMF and the reaction mixture was heated to 80 °C for 10 min. The molar activity of [^18^F]fluoroform was determined based on HPLC analysis of the [^18^F]trifluoromethylated product (Grace Smart C18 5µ 4.6x250mm, acetonitrile/water/TFA 30:70:0.1, 1 mL/min).

Table S4 Radiochemical yield and molar activity depending on the amount of base/complexant; reaction conditions: 10 min, 40 °C, 1 mM triazolium precursor, 1 mL MeCN (0.1% water); n=3.

| Entry | Base  µmol | Complexant  µmol | RCY±SD  % (dc) | MA±SD  % (dc) |
| --- | --- | --- | --- | --- |
| 1 | 0.015 | 0.035 | 0±0 | n.d. |
| 2 | 0.038 | 0.088 | 11±12 | 314 (n=1) |
| 3 | 0.075 | 0.175 | 49±10 | 75±40 |
| 4 | 0.113 | 0.263 | 58±3 | 39±4 |
| 5 | 0.150 | 0.350 | 55±4 | 25±7 |
| 6 | 0.750 | 1.750 | 27±2 | 7±3 |
| 7 | 1.500 | 3.500 | 17±2 | 5±2 |

## Optimisation of other parameters using 5 GBq [^18^F]fluoride

Dry [^18^F]fluoride was obtained as described in in the main manuscript by trapping [^18^F]triflyl fluoride in 890 µL solvent (MeCN, THF or DMF) and 10 µL base complexant stock solution (K_2_CO_3_/K_222_, KHCO_3_/K_222_ or K_2_CO_3_/18-cr-6; see main manuscript). The trapping vial was heated to 40 °C or 80 °C and 100 µL 10 mM triazolium precursor **4** in MeCN was added. After 1 or 10 minutes reaction time [^18^F]fluoroform was purged out of the solution with a helium flow of 10 mL/min. It was led over a silica plus long cartridge and an ascarite column and was trapped at -60 °C in a vessel containing 900 µL DMF and 2 mg (10 µmol) benzophenone. The radiochemical yield of the [^18^F]fluoroform was determined by dividing the decay-corrected radioactivity of the trapped [^18^F]fluoroform in DMF by the radioactivity of reaction solution before distillation. To the same vessel was then added 100 µL of 0.3 M potassium *tert*-butoxide in DMF and the reaction mixture was heated to 80 °C for 10 minutes. Hereafter, the molar activity of [^18^F]fluoroform was determined based on HPLC analysis of the [^18^F]trifluoromethylated product (Grace Smart C18 5µ 4.6x250mm, acetonitrile/water/TFA 30:70:0.1, 1 mL/min).

Table S5 Molar activity and radiochemical yield of [^18^F]fluoroform synthesised from 5 GBq [^18^F]fluoride under different conditions; 990 µL solvent, 10 µL base complexant stock solution, 1 mM triazolium precursor; n = 3

| Entry | Solvent | Base/  complexant | Time  (min) | Temp.  (°C) | RCY±SD  (%, dc) | MA±SD  (%, dc) |
| --- | --- | --- | --- | --- | --- | --- |
| 1 | MeCN | K_2_CO_3_/K_222_ | 10 | 40 | 55±4 | 25±7 |
| 2 | THF | K_2_CO_3_/K_222_ | 10 | 40 | 38±6 | 43±8 |
| 3 | DMF | K_2_CO_3_/K_222_ | 10 | 40 | 10±5 | n.d. |
| 4 | MeCN | KHCO_3_/K_222_ | 10 | 40 | 57±2 | 27±5 |
| 5 | MeCN | K_2_CO_3_/18-cr-6 | 10 | 40 | 26±12 | 42±40 |
| 6 | MeCN | K_2_CO_3_/K_222_ | 1 | 40 | 58±2 | 32±21 |
| 7 | MeCN | K_2_CO_3_/K_222_ | 10 | 80 | 46±3 | 36±13 |

## Optimisation of various parameters using 25 GBq [^18^F]fluoride

Dry [^18^F]fluoride was obtained from 25 GBq aqueous [^18^F] as described in in the main manuscript by trapping [^18^F]triflyl fluoride in MeCN and K_2_CO_3_/K_222_ stock solution. The trapping vial was heated to 40 °C or 80 °C and 100 µL 10 mM triazolium precursor **4** in MeCN was added. After 10 minutes [^18^F]fluoroform was purged out of the solution with a helium flow of 10 mL/min. It was led over a silica plus long cartridge and an ascarite column and was trapped at -60 °C in a vessel containing 900 µL DMF and 2 mg (10 µmol) benzophenone. The radiochemical yield of the [^18^F]fluoroform was determined by dividing the decay-corrected radioactivity of the trapped [^18^F]fluoroform in DMF by the radioactivity of reaction solution before distillation. To the same vessel was then added 100 µL of 0.3 M potassium *tert*-butoxide in DMF and the reaction mixture was heated to 80 °C for 10 minutes. Hereafter, the molar activity of [^18^F]fluoroform was determined based on HPLC analysis of the [^18^F]trifluoromethylated product (Grace Smart C18 5µ 4.6x250mm, acetonitrile/water/TFA 30:70:0.1, 1 mL/min).

Table S6 Molar activity and radiochemical yield of fluoroform synthesised from 25 GBq [^18^F]fluoride under different conditions, t=10 min; * Volume was determined as follows: V(solvent) = 1 mL - (volume of base cryptand stock solution) - (100 µL triazolium solution).

| E. | Temp.  (°C) | Prec.  (µmol) | Base/complexant | | | Solvent  (µL)* | RCY±SD  (%, dc) | MA±SD  (%, dc) | n |
| --- | --- | --- | --- | --- | --- | --- | --- | --- | --- |
|  |  |  | **Base**  **(µmol)** | **Complexant**  **(µmol)** | **Stock**  **solution**  **(µL)** |  |  |  |  |
| 1 | 40 | 1 | 0.075 K_2_CO_3_ | 0.175 K_222_ | 5 | 895 MeCN | 0±0 | n.d. | 2 |
| 2 | 40 | 1 | 0.15 K_2_CO_3_ | 0.35 K_222_ | 10 | 890 MeCN | 25±7 | 67±20 | 3 |
| 3 | 40 | 1 | 0.3 K_2_CO_3_ | 0.7 K_222_ | 20 | 880 MeCN | 38±1 | 57±4 | 2 |
| 4 | 40 | 1 | 0.45 K_2_CO_3_ | 1.05 K_222_ | 30 | 870 MeCN | 35±1 | 30±1 | 2 |
| 5 | 80 | 1 | 0.15 K_2_CO_3_ | 0.35 K_222_ | 10 | 890 MeCN | 40±3 | 102±39 | 4 |
| 6 | 80 | 0.75 | 0.15 K_2_CO_3_ | 0.35 K_222_ | 10 | 890 MeCN | 32±8 | 92±8 | 2 |
| 7 | 80 | 2 | 0.15 K_2_CO_3_ | 0.35 K_222_ | 10 | 890 MeCN | 24±5 | 99±69 | 2 |

## Calculations concerning base consumption during [^18^F]fluoride release from [^18^F]triflyl fluoride

The base has two different roles in the whole synthesis procedure: it releases [^18^F]fluoride from [^18^F]triflyl fluoride and takes part in the [^18^F]fluoroform formation. Since only very low amounts of base are used (e.g. 0.15 µmol K_2_CO_3_), it could be possible that depending on the molar activity of the [^18^F]fluoride the base is already completely consumed during the [^18^F]fluoride release and is not available for the [^18^F]fluoroform formation anymore. This is shown by the following calculations, assuming an average molar activity of [^18^F]fluoroform of 100 GBq/µmol which is a generally obtained molar activity in our institute (Radionuclide Center, Amsterdam UMC).

**Amounts of chemicals:**

**150 nmol K_2_CO_3_**

350 nmol K_222_

1000 nmol triazolium precursor **4**

**Amount of aqueous [^18^F]fluoride at start of synthesis**

Assumption: average A_m_ of [^18^F]fluoride is ~100 GBq/µmol

n = A/ A_m_

A= 0.5 GBq 🡪 n = 5 nmol

A = 5 GBq 🡪 n = 50 nmol

A = 25 GBq 🡪 n = 250 nmol

**[^18^F]triflyl fluoride:**

Roughly 0.7 * amount of aqueous [^18^F]fluoride (see RCY [^18^F]triflyl fluoride in Table S1)

A= 0.5 GBq 🡪 n = 5 nmol * 0.7 = 3.5 nmol

A = 5 GBq 🡪 n = 50 nmol * 0.7 = 35 nmol

**A = 25 GBq 🡪 n = 250 nmol * 0.7 = 175 nmol**

# ^1^H NMR experiments

## Stability of triazolium precursor 4 in presence of K_2_CO_3_ and K_222_


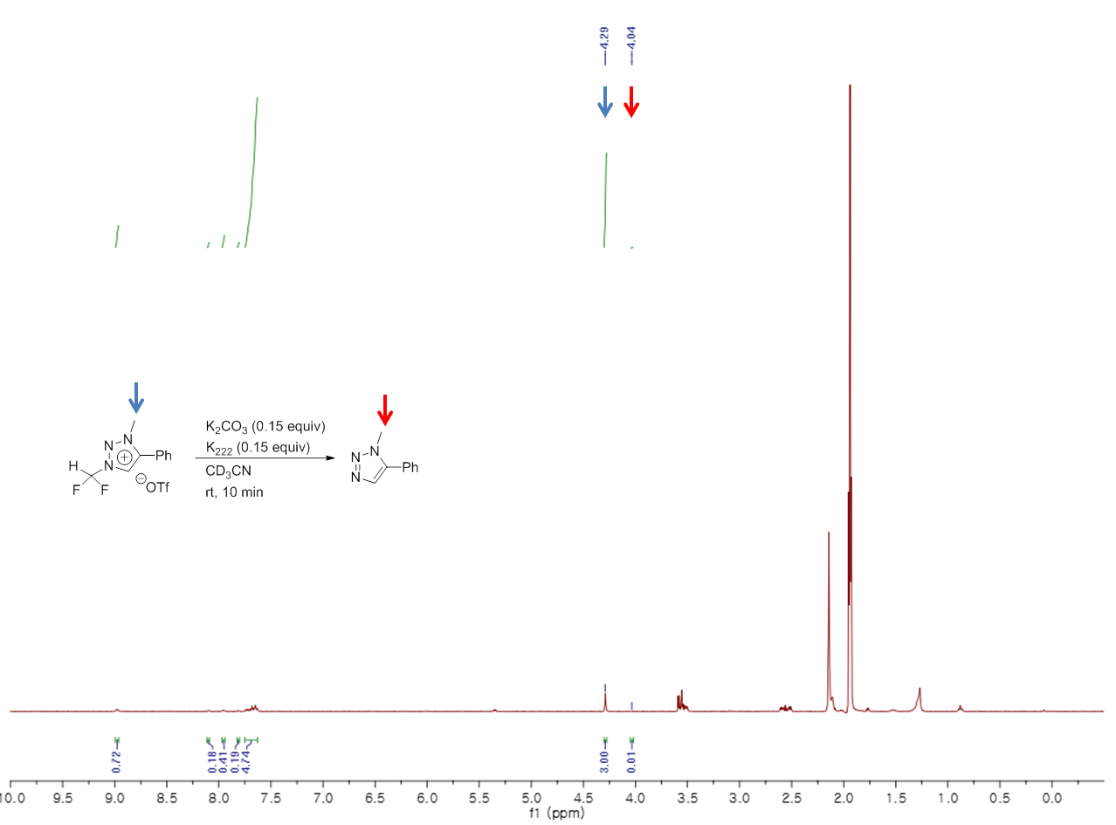


Figure S1 ^1^H NMR spectrum of the reaction mixture (Table 1, entry 1, main manuscript).

**
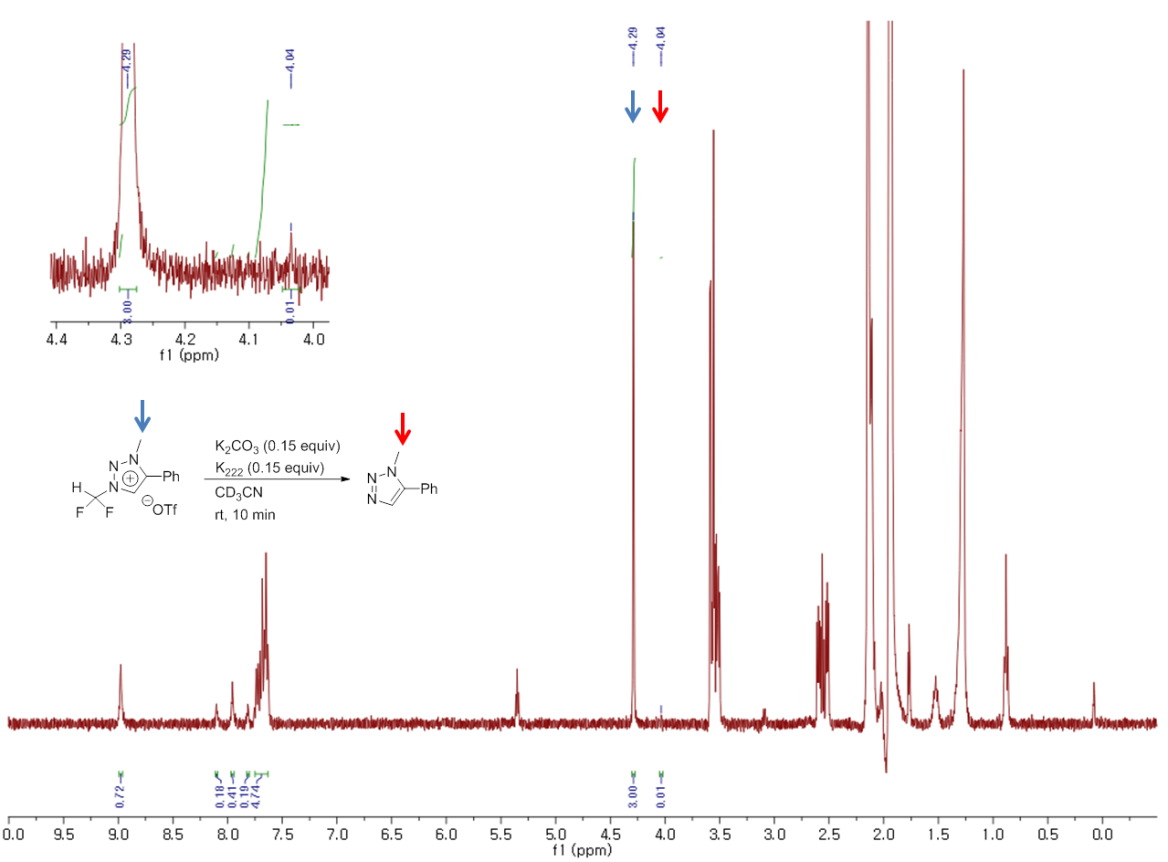
**

Figure S2 Vertical expansion of Figure S1 (Table 1, entry 1, main manuscript).

**
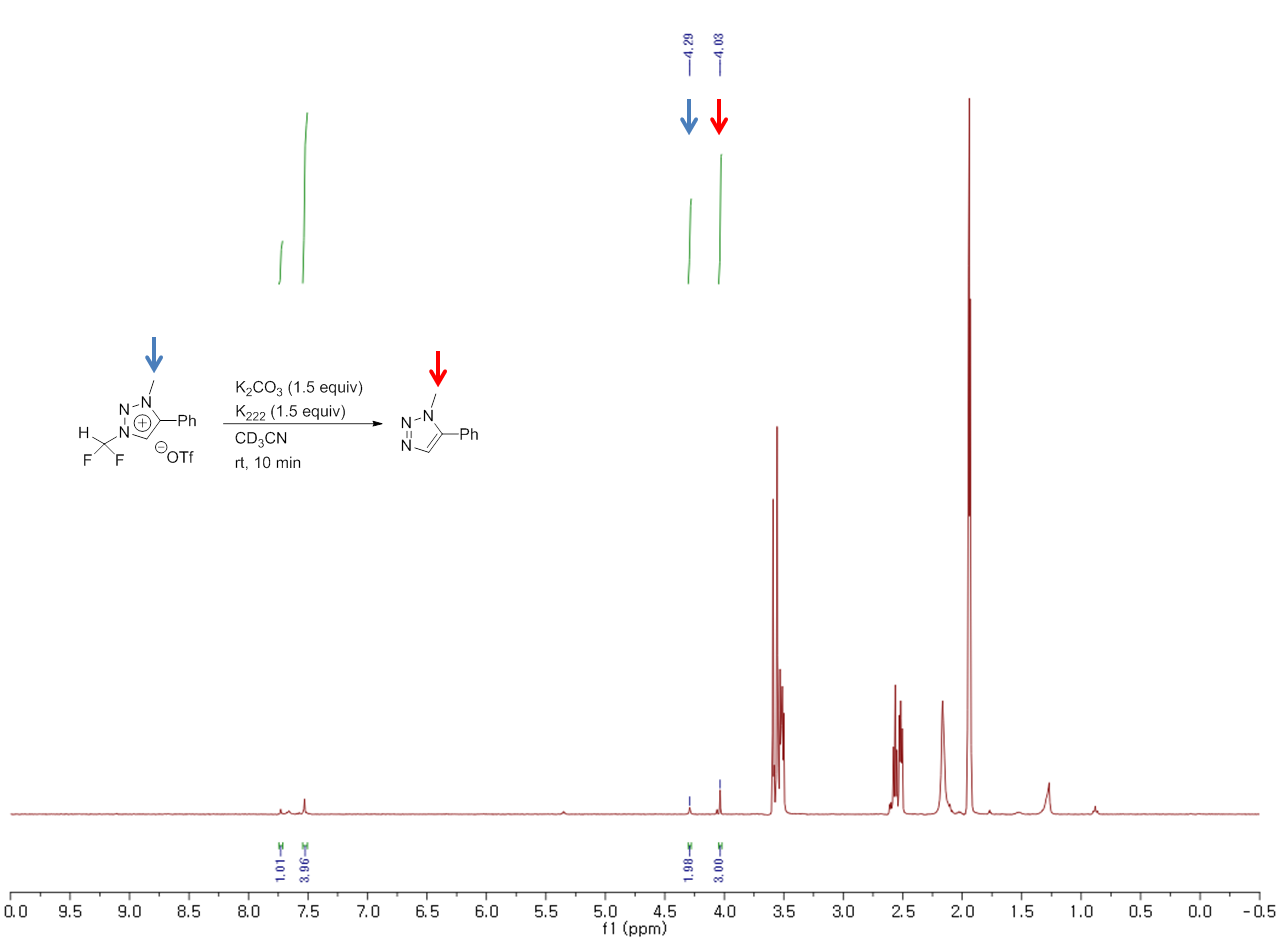
**

Figure S3 ^1^H NMR spectrum of the reaction mixture (Table 1, entry 2, main manuscript).

**
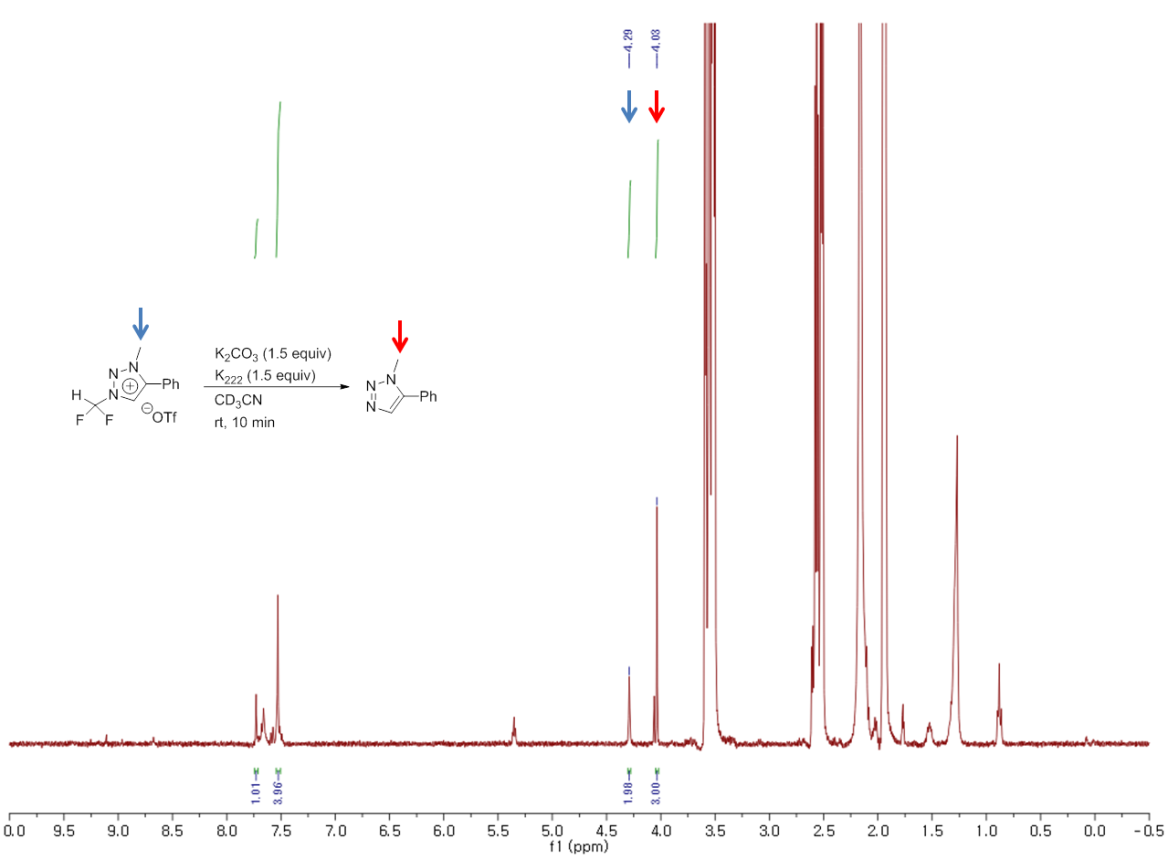
**

Figure S4 Vertical expansion of Figure S3 (Table 1, entry 2, main manuscript).

**
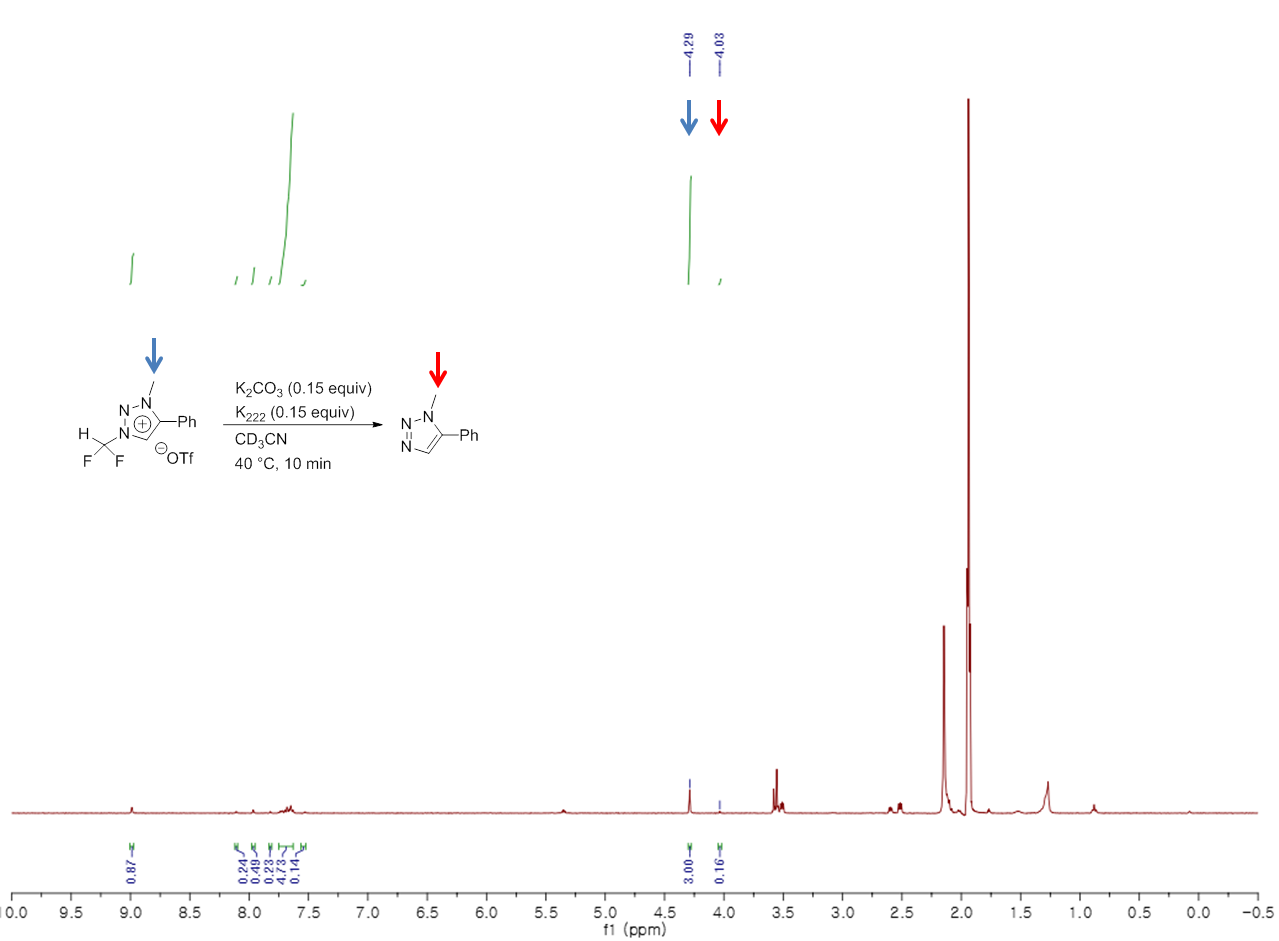
**

Figure S5 ^1^H NMR spectrum of the reaction mixture (Table 1, entry 3, main manuscript).

**
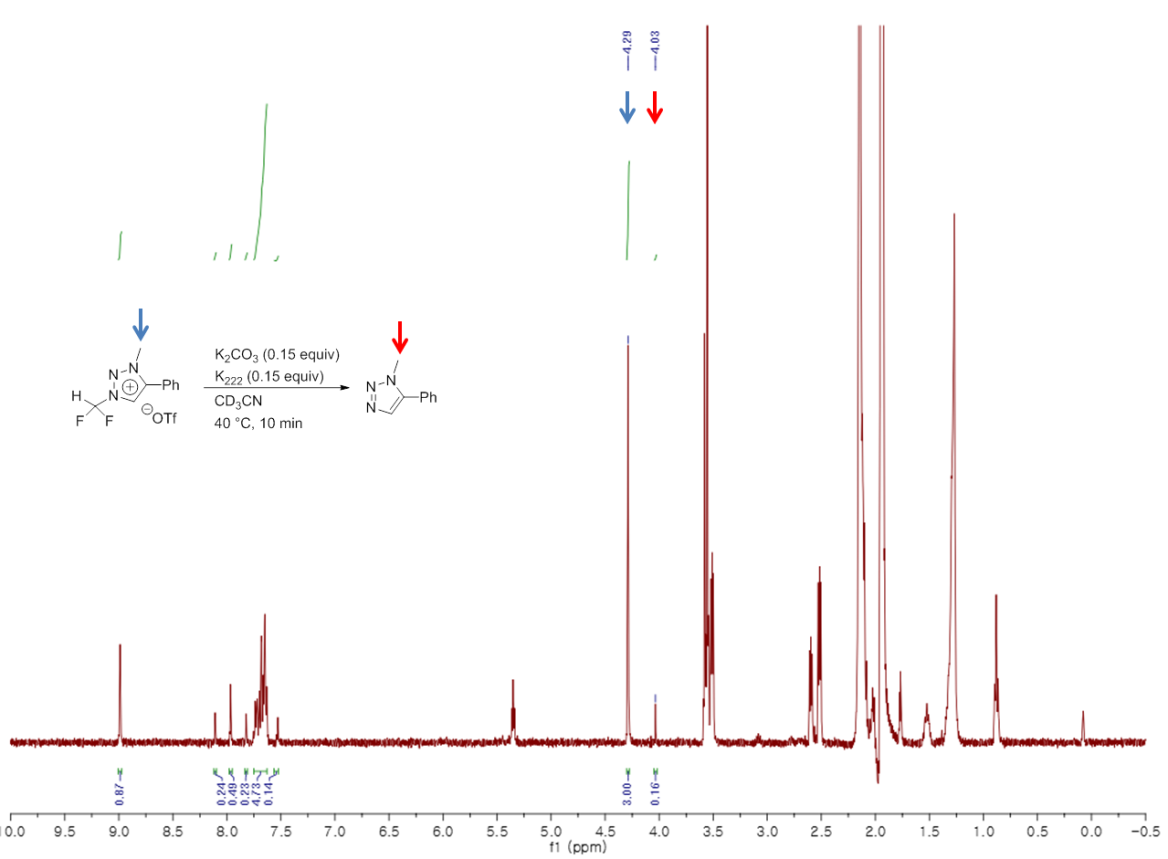
**

Figure S6 Vertical expansion of Figure S5 (Table 1, entry 3, main manuscript).

**
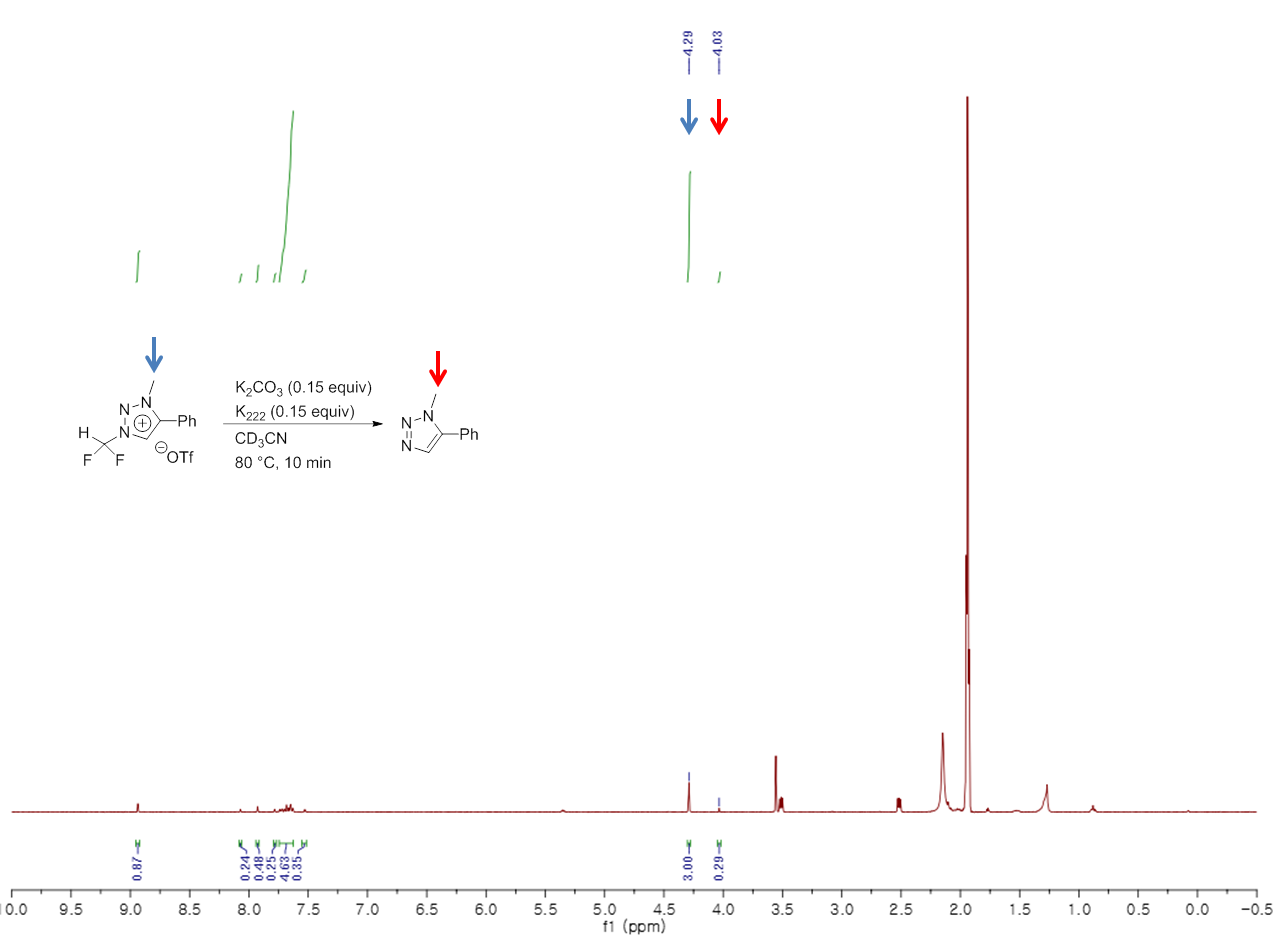
**

Figure S7 ^1^H NMR spectrum of the reaction mixture (Table 1, entry 4, main manuscript).

**
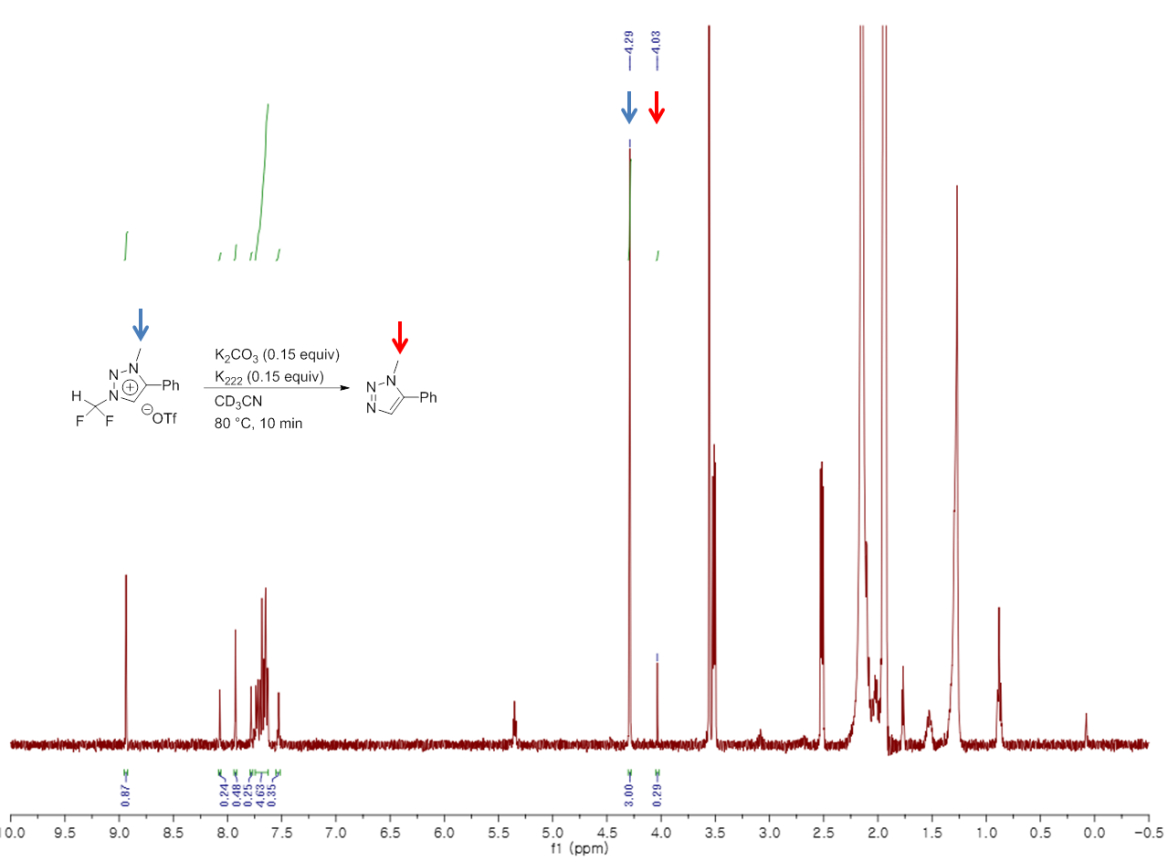
**

Figure S8 Vertical expansion of Figure S7 (Table 1, entry 4, main manuscript).

## ^1^H NMR experiments for determining the reaction order

**
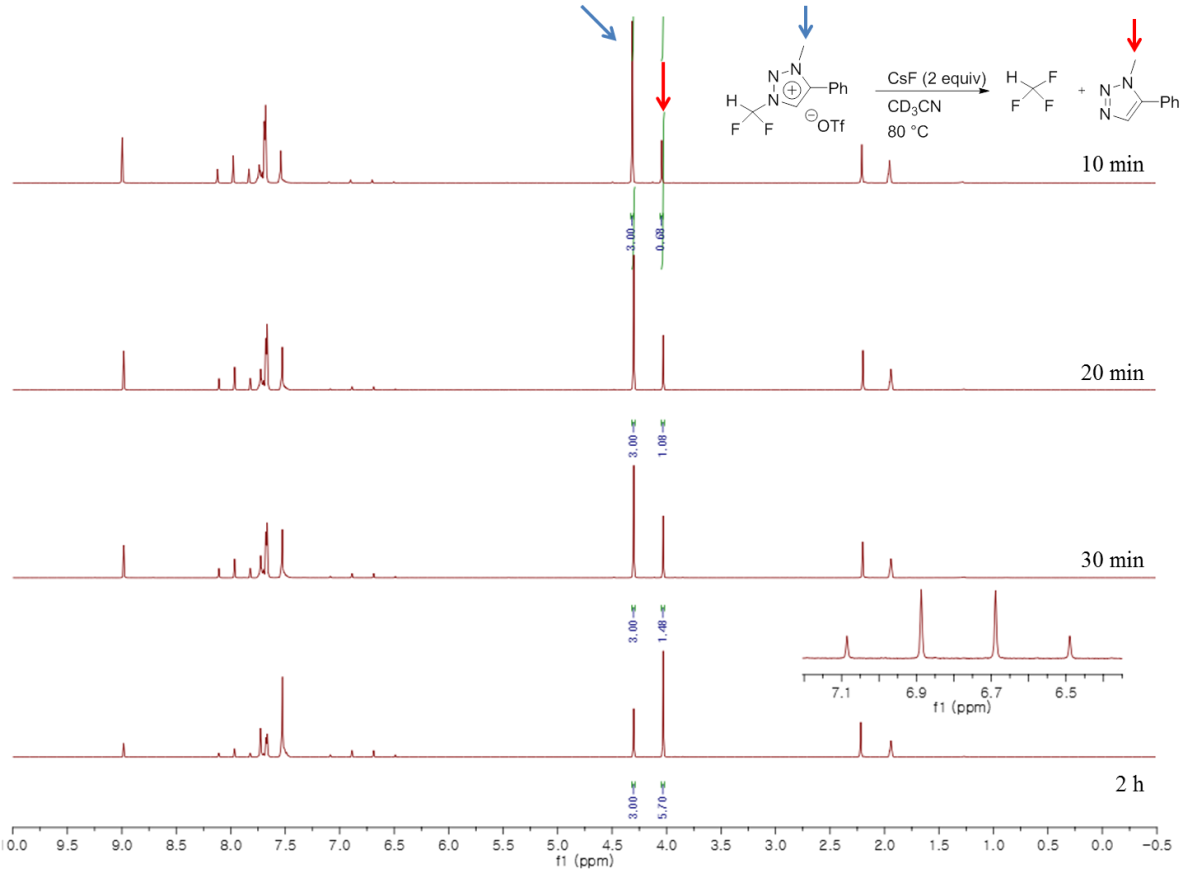
**

Figure S9 ^1^H NMR spectra of the reaction mixture (Table 5, 10 min–2 h, main manuscript).

**
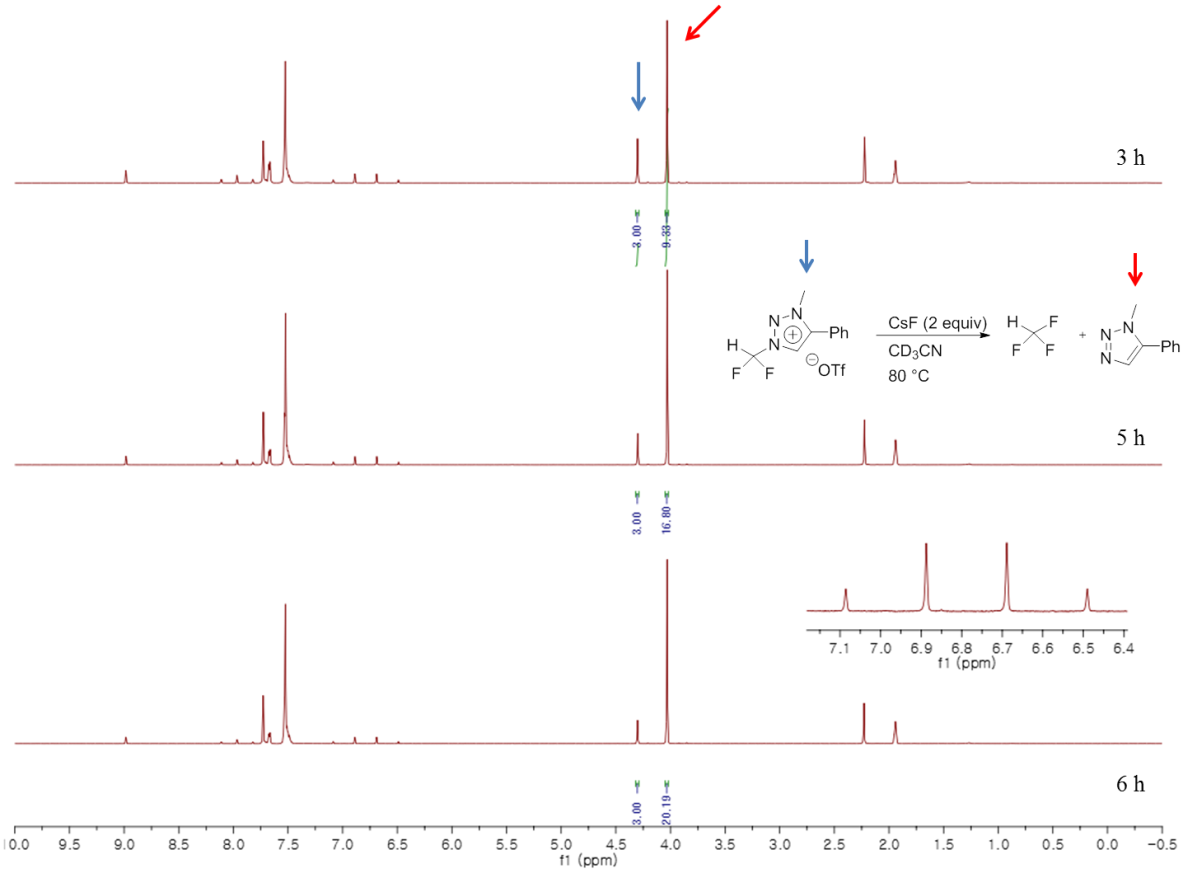
**

Figure S10 ^1^H NMR spectra of the reaction mixture (Table 5, 3 h–6 h, main manuscript).

# HPLC chromatograms

## **1-(Difluoromethyl)-3-methyl-4-phenyl-1*H*-1,2,3-triazol-3-ium triflate**


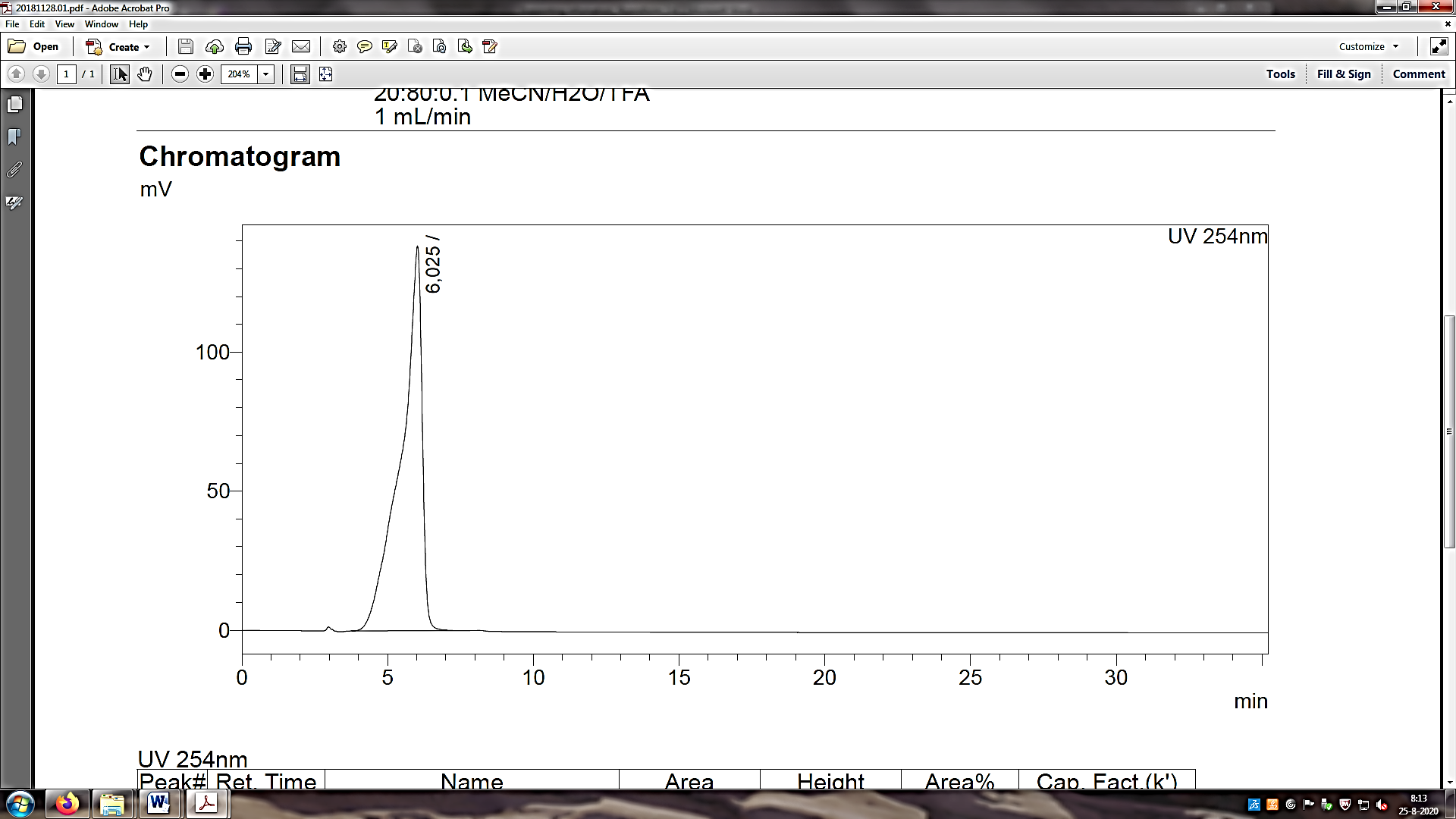


Figure S11 HPLC chromatogram of the triazolium precursor 4 (6.025 min), 0.01 M; Column: Alltima C18 5µ 4.6x250mm, Eluent: 20:80:0.1 MeCN/H_2_O/TFA; 40 µL injected.

## α-[^18^F](Trifluoromethyl)benzhydrol


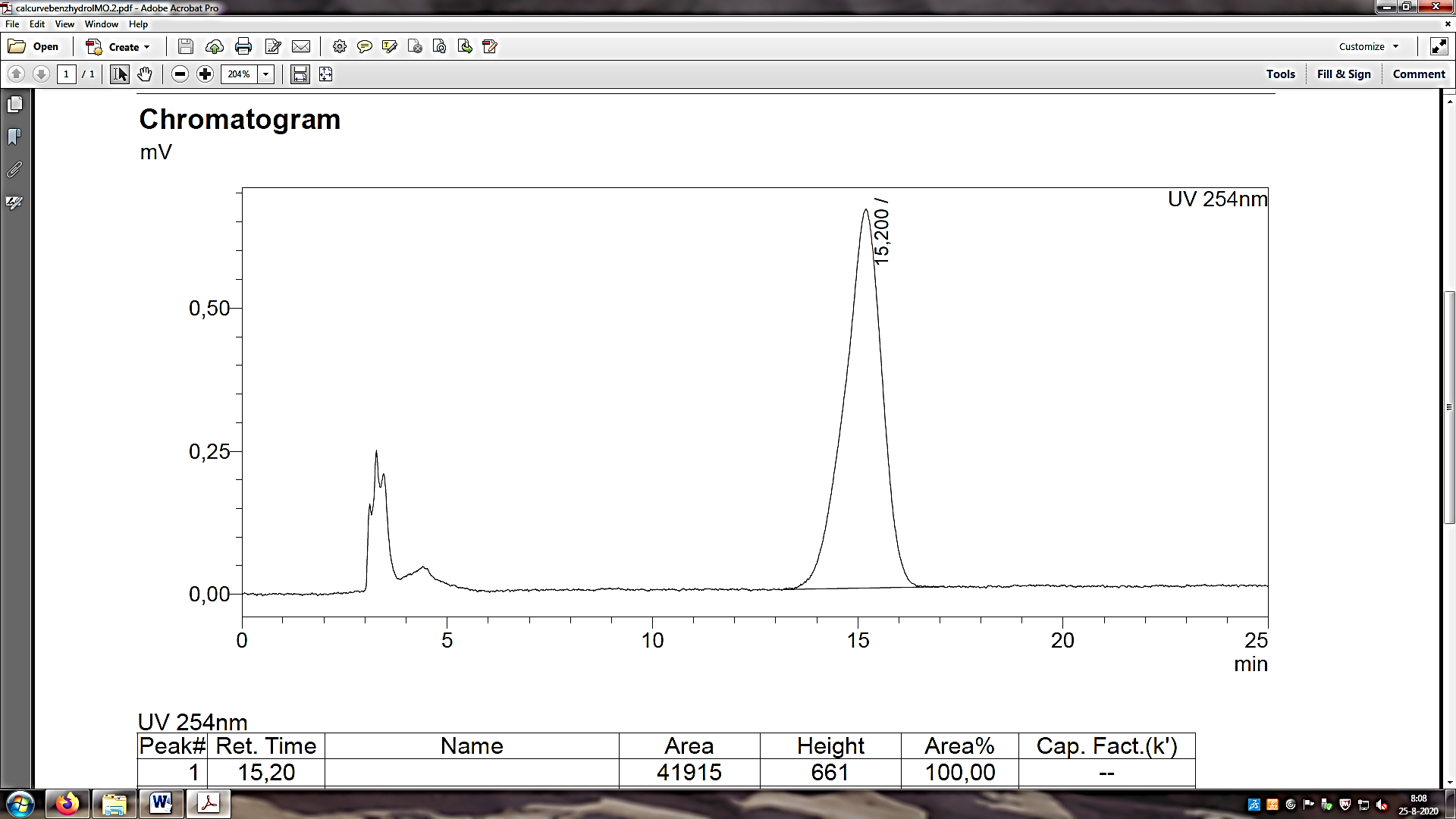
Figure S12 HPLC chromatogram of α-(trifluoromethyl)benzhydrol (15.2 min); Column: Grace Smart C18 5µ 4.6x250mm, Eluent: 30:70:0.1 MeCN/H_2_O/TFA.

## Reaction mixture


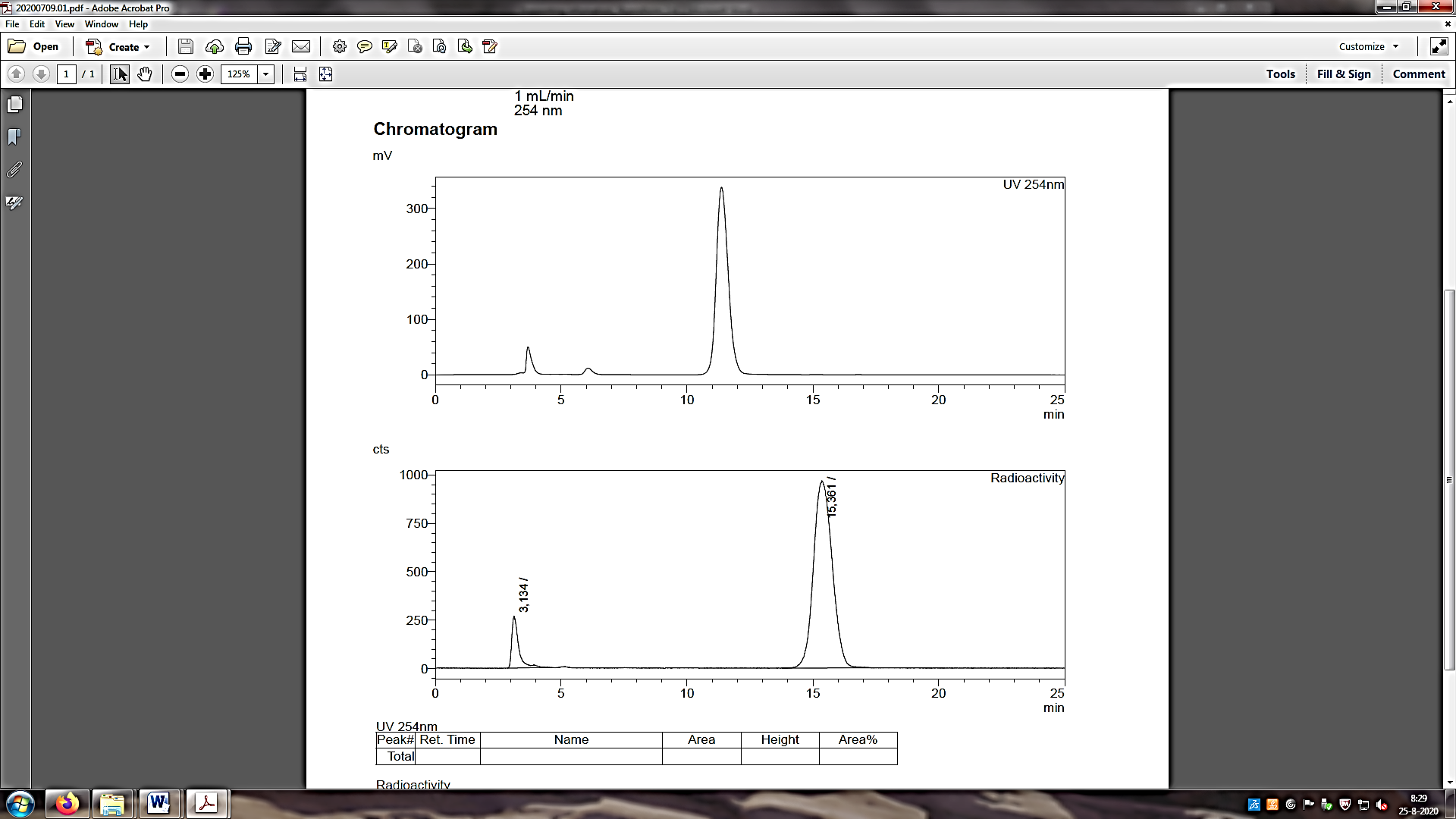


Figure S13 HPLC chromatogram of the reaction mixture of the [^18^F]trifluoromethylation of benzophenone, 2 µL injected; Top: UV at 254 nm; bottom: radioactivity measurement, peak at 15.4 min is α-[^18^F](trifluoromethyl)benzhydrol; Column: Grace Smart C18 5µ 4.6x250mm, Eluent: 30:70:0.1 MeCN/H_2_O/TFA.


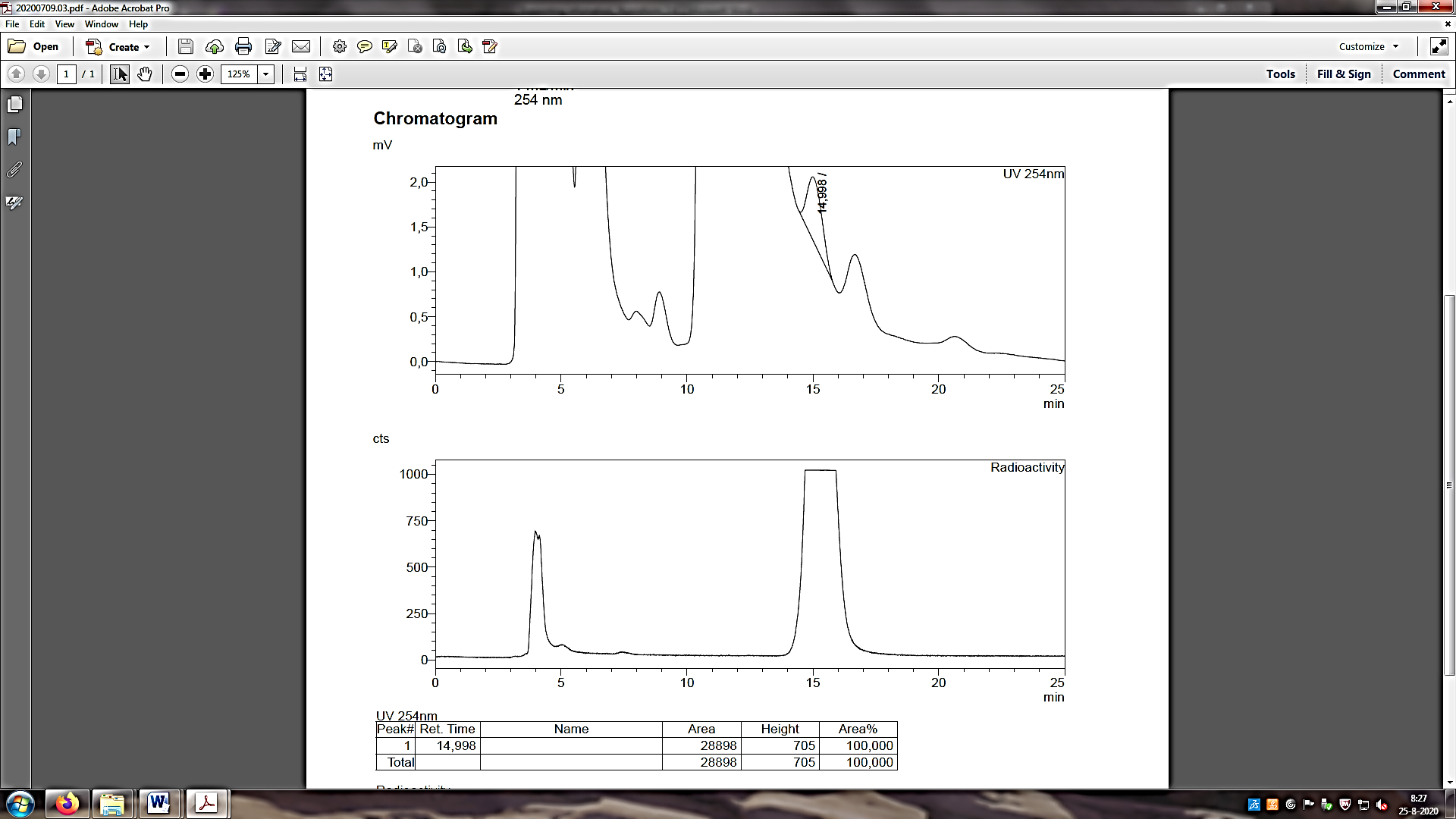


Figure S14 HPLC chromatogram of the reaction mixture of the [^18^F]trifluoromethylation of benzophenone, 20 µL injected; Top: UV at 254 nm, peak at 15.0 min is α-(trifluoromethyl)benzhydrol; bottom: radioactivity measurement; Column: Grace Smart C18 5µ 4.6x250mm, Eluent: 30:70:0.1 MeCN/H_2_O/TFA.

# 
