# Supplementary material for: Evaluating N‐difluoromethyltriazolium triflate as a precursor for the synthesis of high molar activity [18F]fluoroform
Source: J Labelled Comp Radiopharm. 2021 Sep 20;64(12):466–76. doi: 10.1002/jlcr.3939 (PMC9293032; doi:10.1002/jlcr.3939)
Supplement: Supplementary file 2 — Supporting information item [file JLCR-64-466-s001.docx]

Evaluating *N*-difluoromethyltriazolium triflate as a precursor for the synthesis of high molar activity [^18^F]fluoroform

Anna Pees^1^, Maria J.W.D. Vosjan^2^, Jin Young Chai^3^, Hyojin Cha^3^, Dae Yoon Chi^3^, Albert D. Windhorst^1^ and Danielle J. Vugts^1^

*^1^ Amsterdam UMC, VU University, Radiology and Nuclear Medicine, Radionuclide Center, De Boelelaan 1085c, Amsterdam, The Netherlands.*

*^2^ BV Cyclotron VU, De Boelelaan 1081,1081 HV Amsterdam, The Netherlands.*

*^3^ Department of Chemistry, Sogang University, 35 Baekbeomro Mapogu, Seoul 04107, Korea.*

Keywords:

[^18^F]fluoroform, high molar activity, triazolium precursor, fluorine-18, [^18^F]trifluoromethylation

# Abstract

The trifluoromethyl group is a prominent motif in biologically active compounds and therefore of great interest for the labelling with the positron emitter fluorine-18 for positron emission tomography (PET) imaging. Multiple labelling strategies have been explored in the past, however most of them suffer from low molar activity due to precursor degradation. In this study the potential of 1-(difluoromethyl)-3-methyl-4-phenyl-1*H*-1,2,3-triazol-3-ium triflate as precursor for the synthesis of the [^18^F]trifluoromethylation building block [^18^F]fluoroform with high molar activity was investigated. The triazolium precursor was reacted under various conditions with [^18^F]fluoride, providing [^18^F]fluoroform with radiochemical yields (RCY) and molar activities (A_m_) comparable and even superior to already existing methods. Highest molar activities (A_m_ = 153±14 GBq/µmol, dc, EOS) were observed for the automated procedure on the Neptis® perform module. Due to its easy handling and good RCY and A_m_ in the [^18^F]fluoroform synthesis the triazolium precursor is a valuable alternative to already known precursors.

# Introduction

Despite the fact that naturally occurring fluorine-containing molecules are rare, fluorine is a very commonly used element in drug design.^1^ This is due to its ability to positively influence the characteristics of a given molecule, e.g. its p*K*_a_, lipophilicity or pharmacokinetics.^1–3^ Furthermore, fluorine and (poly)fluorinated groups can act as a bioisostere for hydrogen and many functional groups such as carbonyl, hydroxyl and nitrile.^2,4^ Besides the fluorine atom, the trifluoromethyl group is one of the most commonly used fluorine-containing structures.

The *in vivo* evaluation of a drug candidate by positron emission tomography (PET), a non-invasive imaging technique, is an emerging process in drug development.^1,5^ It relies on biologically active molecules that are labelled with positron-emitting radionuclides, so-called PET tracers.^5^ Of these radionuclides, fluorine-18 is one of the most popular ones. It has a convenient half-life (110 min) and excellent beta decay characteristics.^4,6^ Also, the role of this element in drug design contributes to the popularity of fluorine-18 as it allows the radiolabelling of many compounds without variation of the original structure.

Among the different radiofluorination strategies, the introduction of radiolabelled CF_3_-groups into potential tracer molecules via [^18^F]trifluoromethylation has gained increasing interest over the past years. Most of the reported methods are based on the use of the ^18^F-labelled building block [^18^F]fluoroform.^7^ Different precursors have been explored, e.g. methyl chlorodiﬂuoroacetate **1**, difluoroiodomethane **2** and the difluoromethylsulfonium salt **3** (Figure 1).^8–12^ However, yielding [^18^F]trifluoromethylated products with high molar activities has proven to be challenging. This is because the precursors decompose under the standard basic radiofluorination conditions releasing [^19^F]fluoride which competes with [^18^F]fluoride in the formation of [^18^F]fluoroform resulting in high amounts of non-labelled compound. Among the above mentioned precursors **1-3**, only difluoroiodomethane **2** provided [^18^F]fluoroform with average molar activities suitable for PET tracer synthesis so far (A_m_ = 97±20 GBq/µmol, n = 3).^13^ This precursor **2** is gaseous and therefore certain handling protocols need to be followed. Furthermore, molar activities still did not reach the levels of usual [^18^F]fluorination reactions (0.1-100 GBq/µmol vs >100 GBq/µmol in standard radiofluorination). As an alternative, a synthetic strategy via [^18^F]fluoromethane and subsequent gas phase fluorination has been developed but on average only moderate molar activities were obtained (A_m_=38±35 GBq/µmol (n = 20) with max. A_m_ = 163 GBq/µmol).^14^ The synthesis of triazolium salt **4** (1-(difluoromethyl)-3-methyl-4-phenyl-1*H*-1,2,3-triazol-3-ium triflate) has recently been reported in literature and its reaction with [^19^F]fluoride has been extensively studied, indicating that this precursor would be able to provide high molar activity [^18^F]fluoroform (Figure 1).^15^ Therefore, our aim was to investigate radioactive reactions of triazolium salt **4** with [^18^F]fluoride to explore whether the triazolium salt **4** would be suitable as precursor for the synthesis of [^18^F]fluoroform and would enable higher radiochemical yields (RCYs) and higher molar activities (A_m_) than previously reported methods.

# Experimental

## General methods and materials

All the chemicals were purchased from commercial sources and used without further purification. ^1^H NMR spectra were recorded on a Varian 400 MHz NMR spectrometer. ^1^H NMR chemical shifts were determined relative to CD_3_CN at δ 1.94 ppm. All radioactive reactions and products were analysed with high-performance liquid chromatography (HPLC) using a Shimadzu SPD-20A system and LabSolutions 5.85 software (Shimadzu Corporation, Japan) with a Grace Smart C18 column (5µ 4.6x250mm), MeCN/H_2_O/TFA 30:70:0.1 or an Alltima C18 (5µ 4.6x250mm), MeCN/H_2_O/TFA 20:80:0.1 as eluent and a flow of 1 mL∙min^-1^. UV active compounds were detected at 254 nm. Radioactive products were identified by comparison with the unlabelled reference compounds. Radioactivity was quantified with a Veenstra VDC-304 dose calibrator.

## Synthesis of triazolium precursor **4**

1-(Difluoromethyl)-3-methyl-4-phenyl-1*H*-1,2,3-triazol-3-ium triflate **4** was prepared in five steps from ethyl bromodifluoroacetate **5** following literature procedures.^15,16^

## Investigation of the stability of precursor **4** in presence of K_2_CO_3_ and K_222_ by ^1^H NMR experiments

### K_2_CO_3_ stock solution

CD_3_OD (2 mL) was added to ground K_2_CO_3_ (2.1 mg, 15 µmol) in a 4 mL vial containing a stirring bar. The solution was stirred until the salt was completely dissolved.

### K_222_ stock solution

CDCl_3_ (0.8 mL) was added to K_222_ (5.3 mg, 14 µmol) in a 4 mL vial.

### Precursor **4** stock solution

CDCl_3_ (0.8 mL) and CD_3_OD (0.4 mL) were added to precursor **4** (7.2 mg, 20 µmol) in a 4 mL vial.

### General procedure for Table 1, entry 1

An aliquot of the K_2_CO_3_ stock solution (10 µL, 0.075 µmol) was added to an NMR tube. CD_3_OD (0.2 mL) was added to the NMR tube. The solvent in the NMR tube was evaporated using a flow of Argon. An aliquot of the K_222_ stock solution (10 µL, 0.175 µmol) was added to a 4 mL vial (vial A) and the solvent was removed under vacuum. An aliquot of precursor **4** stock solution (30 µL, 0.5 µmol) was added to a second 4 mL vial (vial B) and the solvent was removed under vacuum. CD_3_CN (0.15 mL) was added to K_222_ in vial A, and the solution in vial A was added to K_2_CO_3_ in the NMR tube. Vial A was rinsed with CD_3_CN (0.1 mL), and the rinsing solution was added to the NMR tube. CD_3_CN (0.15 mL) was added to precursor **4** in vial B, and the solution in vial B was added to the NMR tube. Vial B was rinsed with CD_3_CN (0.1 mL), and the rinsing solution was added to the NMR tube. The NMR tube was sealed with a cap, and after 10 min at rt, a ^1^H NMR of the mixture was recorded. Note: When CD_3_OD (0.2 mL) was added to the NMR tube, the solvent was added with the syringe tip touching the side of the NMR tube to move K_2_CO_3_ remaining on the side of the NMR tube to the bottom of the NMR tube.

**Procedure for Table 1, entry 2:** The same procedure as for entry 1 was used, except that the amounts of base and complexant were 10x higher: K_2_CO_3_ stock solution (100 µL, 0.75 µmol) and K_222_ stock solution (100 µL, 1.75 µmol) were used.

**Procedure for Table 1, entry 3:** The same procedure as for entry 1 was used, except that the reaction mixture was heated: the NMR tube was sealed with a cap, and after 10 min at 40 °C, the NMR tube was cooled to 0 °C. ^1^H NMR of the mixture was recorded.

**Procedure for Table 1, entry 4:** **:** The same procedure as for entry 1 was used, except that the reaction mixture was heated: the NMR tube was sealed with a cap, and after 10 min at 80 °C, the NMR tube was cooled to 0 °C. ^1^H NMR of the mixture was recorded.

NMRs can be found in the SI, Figure S1 to S8.

## Procedure for determining the reaction order by ^1^H NMR experiments

CD_3_CN (0.15 mL) was added to CsF (25.5 mg, 0.168 mmol) in an NMR tube. CD_3_CN (0.6 mL) was added to precursor **4** (30.0 mg, 0.084 mmol) in a 20 mL vial, and then the solution of **4** was transferred to the NMR tube. The NMR tube was sealed with a cap, and after the reaction time at 80 °C, a ^1^H NMR of the mixture was recorded (reaction times: 10 min, 20 min, 30 min, 2 h, 3 h, 5 h and 6 h). NMRs can be found in the SI, Figure S9 and S10.

## Radiochemistry

### Base-complexant stock solutions

150 µmol (20 mg) K_2_CO_3_ or 300 µmol (30 mg) KHCO_3_, and 350 µmol complexant (93 mg 18-crown-6 or 130 mg kryptofix 222) were dissolved in 9 mL acetonitrile and 1 mL water.

### Dry [^18^F]fluoride via [^18^F]triflyl fluoride

[^18^F]Fluoride was produced by irradiation of an ^18^O-enriched water target and was trapped on a Chromafix^®^ 30-PS-HCO_3_ cartridge (Macherey-Nagel, Germany). It was eluted from the cartridge with 500 µL 0.1 M aqueous potassium sulfate solution. The eluate was collected in a vessel containing 850 µL DMF and 150 µL 0.1 M *N,N*-bis(trifluoromethylsulfonyl)aniline in DMF was added. The vessel was heated to 40 °C and the gaseous [^18^F]triflyl fluoride was purged out of the reaction mixture with a flow of helium (10 mL/min) for about 5 minutes. The [^18^F]triflyl fluoride was distilled over phosphorus pentoxide and trapped in a second reaction vessel containing a certain volume of base complexant stock solution (**x**, volume depending on the amounts of base and complexant; base: 0.015-1.5 µmol K_2_CO_3_ or 0.3 µmol KHCO_3_, complexant: 0.035-3.5 µmol K_222_ or 0.35 µmol 18-crown-6) and 900-**x** µL solvent (MeCN, THF, DMF). [^18^F]Fluoride was released in presence of the base and complexant.

The RCY of dry [^18^F]fluoride was determined by dividing the decay-corrected radioactivity of the distillate by the radioactivity of the reaction solution before distillation. An overview of the RCYs can be found in the SI (Table S1).

### [^18^F]Fluoroform synthesis

Dry [^18^F]fluoride was obtained from 25 GBq aqueous [^18^F]fluoride as described under section 2.5.2 by trapping gaseous [^18^F]triflyl fluoride in a solution of 10 µL K_2_CO_3_/K_222_ stock solution in MeCN/H_2_O (see 2.5.1) and 890 µL MeCN. After the trapping solution was heated to 80 °C, 100 µL 10 mM triazolium precursor **4** (1 µmol) in MeCN was added and the mixture reacted for 10 minutes at 80 °C. [^18^F]Fluoroform was purged out of the solution with a flow of helium (10 mL/min) for about 3 minutes, led over a Waters Sep-Pak® Plus Silica cartridge (long) and trapped in 1 mL DMF cooled to -60 °C.

The RCY of [^18^F]fluoroform was determined by dividing the decay-corrected radioactivity of the distillate by the radioactivity of the reaction mixture before distillation.

For exemplary HPLC chromatograms of the analysis of the [^18^F]fluoroform reaction see SI, Figure S11-14.

### Automated [^18^F]fluoroform synthesis on the ORA Neptis^®^ synthesizer

[^18^F]Fluoroform was synthesized starting from 25 GBq [^18^F]fluoride with the triazolium precursor **4** on the ORA Neptis^®^ synthesizer according to the procedure described in literature.^13^ Exactly the same setup and sequence as for CHIF_2_ were used, except for two changes: 100 µL 10 mM triazolium precursor **4** (1 µmol) in MeCN was used instead of CHIF_2_. The heating period of the oven of the synthesizer was prolonged to 2.5 min to ensure that a reaction temperature of 80 °C was reached.

# Results and Discussion

## Stability of precursor **4** in presence of K_2_CO_3_ and K_222_

In contrast to difluoroiodomethane **2**, triazolium salt **4** is not commercially available. **4** was prepared according to literature procedures and was obtained as a white solid with a melting point of 104–109 °C (Scheme 1).^15,16^

The stability of precursor **4** in pure solvent (MeCN, DMF) at elevated temperatures was studied thoroughly in the literature: when **4** was heated to 80 °C for 60 h in acetonitrile or under reflux in DMF (bp = 153 °C) for 1 h, no decomposition was observed.^15^ However, since difluoroiodomethane proved to be particularly unstable under the basic radiofluorination conditions,^13^ we investigated the stability of precursor **4** in presence of K_2_CO_3_/K_222_ (Table 1). When using 0.15 eq. K_2_CO_3_ at room temperature (Table 1, entry 1) only a trace amount (0.3 %) of dedifluoromethylated triazole **10** was detected and 99.7 % of the precursor remained intact. Increasing the amount of base to 1.5 eq. led to significantly more degradation (60% methyl triazole **10**, entry 2). Also an increase of the temperature promoted degradation: the percentage of methyl triazole **10** in presence of 0.15 eq. K_2_CO_3_ increased to 5 % at 40 °C and 9 % at 80 °C (entries 3 and 4). We therefore concluded that as a consequence of this instability, low amounts of base and complexant are key for obtaining [^18^F]fluoroform with high molar activity, as is the case with difluoroiodomethane.^13^

## Radiochemistry

Under standard radiofluorination conditions (azeotropic drying; 15 µmol K_2_CO_3_, 35 µmol K_222_) the reaction of triazolium precursor **4** to [^18^F]fluoroform proceeded with a RCY of 61±5 % (n=3) and a molar activity of 0.5±0.1 GBq/µmol (n = 3), which is comparable to data obtained with difluoroiodomethane as precursor.^9,10^ The molar activity of [^18^F]fluoroform **11** was determined by formation of the UV active [^18^F]trifluoromethylated product **15** (Scheme 2). Based on previous data with difluoroiodomethane as precursor^13^ and the stability data obtained with triazolium precursor **4** (Table 1), the amounts of base and complexant were decreased 100-fold and the effect on the molar activity and RCY investigated. The molar activity obtained with triazolium precursor **4** could be improved to 102±39 GBq/µmol while still having good RCY of 40±3 % (n = 4), which was higher than the results previously obtained with difluoroiodomethane (RCY = 35±4 %, A_m_ = 78±38 GBq/µmol)^13^ under same reaction conditions (25 GBq starting activity, 80 °C, 10 min, 1 µmol precursor, 0.15 µmol K_2_CO_3_, 0.35 µmol K_222._, 1 mL MeCN).

Encouraged by these results, we studied the [^18^F]fluoroform formation reaction with triazolium precursor **4** in more detail. To have full flexibility for the screening of the reaction conditions, the [^18^F]triflyl fluoride method was used to produce dry [^18^F]fluoride (see Scheme 2).^17^ [^18^F]Triflyl fluoride was generated by radiofluorination of bistriflate precursor **12** under aqueous conditions and was distilled into various solvents (MeCN, THF, DMF; trapping temperatures according to melting point). [^18^F]Fluoride was released in presence of various types and amounts of bases (K_2_CO_3_ and KHCO_3_) and complexants (K_222_ and 18-crown-6). The RCY of [^18^F]fluoride depending on solvents, bases and complexants can be found in Table S1 in the supporting information (SI). For all conditions good to excellent RCYs of dry [^18^F]fluoride were obtained.

To optimize the RCY and molar activity of the subsequent [^18^F]fluoroform formation with triazolium precursor **4**, the following reaction parameters were evaluated: 1) reaction temperature, 2) type and amounts of base and complexant, 3) precursor amount, 4) reaction time and 5) solvent. In the following discussion, all reported RCYs are calculated from dry [^18^F]fluoride.

The reaction temperature proved to be an important parameter for the optimal [^18^F]fluoroform formation. The optimization reactions were carried out with about 500 MBq of dry [^18^F]fluoride, 1 µmol of triazolium precursor **4**, 0.15 µmol of potassium carbonate and 0.35 µmol of kryptofix 222. Under these conditions 40 °C was the optimal reaction temperature, resulting in RCYs of 52±6 % (dc, n=3) (see Figure 2 and SI, Table S2).

In the subsequent reactions starting with higher amounts of [^18^F]fluoride however, the temperature optimum shifted towards higher temperatures: with 5 GBq [^18^F]fluoride the RCYs at 80 °C were almost as high as at 40 °C and with 25 GBq [^18^F]fluoride 80 °C was clearly preferred (see Table 2).

Next, the effect of the amount of potassium carbonate and kryptofix 222 on the RCY and molar activity was investigated. An overview of the results using 5 GBq [^18^F]fluoride is given in Table 3 and Table S4 of the SI. The results are in line with our previous results using difluoroiodomethane as precursor: the lower the amount of base, the higher the molar activity.^13^ However, variation of the amount of potassium carbonate and kryptofix 222 had a much larger effect on the RCY observed for triazolium precursor **4** than observed for difluoroiodomethane at their optimal reaction temperatures (40 °C and 80 °C, respectively). Optimal results were obtained in the small range of 0.075-0.150 µmol potassium carbonate, outside this range the yields significantly dropped and at very low base amounts (0.015 µmol) no product was formed at all. With 0.038 µmol potassium carbonate, a very high molar activity of 314 GBq/µmol was obtained but the A_m_ could only be determined once due to low RCYs (see Table 3, entry 2). Although this is N=1, it is an interesting observation.

Changing from 5 GBq starting activity to 25 GBq resulted in a shift in the optimal base amount for obtaining good RCYs and molar activities. With 0.075 µmol potassium carbonate, 25 GBq of [^18^F]fluoride and 40 °C reaction temperature no [^18^F]fluoroform was formed. Only when the amount was increased to 0.30-0.45 µmol potassium carbonate (see Table 4) acceptable RCYs with low standard deviation could be obtained. Using this amount of base, molar activities were however lower.

Good RCYs at low base amounts (0.15 µmol K_2_CO_3_, 0.35 µmol kryptofix 222) and in consequence high molar activities could be restored by performing the reaction at 80 °C: [^18^F]fluoroform could be synthesized with a RCY of 40±3 % and a molar activity of 102±39 GBq/µmol (n=4) (Table 2, entry 6).

Based on the evaluation of the two parameters, reaction temperature and amounts of base and complexant, as described above, it can be concluded that low amounts of base lead to less precursor degradation and therefore high molar activity [^18^F]fluoroform. However, a certain amount of base is needed to enable the [^18^F]fluoroform formation at 40 °C (see Table 3). When higher amounts of radioactivity are used (see Table 4) more base is consumed for the release of the [^18^F]fluoride from [^18^F]triflyl fluoride and as a consequence less base is available for the subsequent [^18^F]fluoroform formation. Therefore, more base needs to be added to obtain comparable RCYs with high amounts of [^18^F]fluoride.

The influence of the temperature is not yet fully understood. Our data show that high temperatures encourage precursor degradation, forming the dedifluoromethylated compound (see Table 1). This could explain the lower reaction temperature optimum that we found for low radioactivity levels (500 MBq [^18^F]fluoride): at 40 °C the radiofluorination still proceeds well while precursor degradation is minimal. Assuming that at high radioactivity levels (25 GBq [^18^F]fluoride) and low amounts of base (0.15 µmol) the base is almost completely consumed during the release of [^18^F]fluoride from [^18^F]triflyl fluoride (see calculations in SI), it might be that under nearly base-free conditions there are few side reactions competing with the radiofluorination reaction and the reaction temperature can be increased to improve the RCYs of the [^18^F]fluoroform formation. However, more in-depth investigations are necessary to support this explanation which is outside the scope of this manuscript.

Subsequently, the influence of the amount of precursor **4** on RCY and molar activity was investigated (see Figure 3 and SI, Table S3). The screening of amounts between 0.2 and 10 µmol with low starting activities (~500 MBq dry [^18^F]fluoride) showed that the highest RCYs (~45 %) could be obtained at precursor amounts of 0.75-2 µmol. Lower amounts of 0.2 µmol still resulted in an acceptable RCY of 34±5 %, whereas the RCY dropped dramatically at high precursor amounts (2±0 % at 10 µmol).

Based on these results the effect of the precursor amount on molar activity was investigated with 25 GBq starting activity by comparing 0.75 µmol with 1 µmol precursor amount (see Table S6 in SI), assuming that reducing the precursor amount might reduce the amount of ^19^F^-^ competing in the reaction and therefore lead to higher molar activities. However, this hypothesis could not be confirmed. Similar molar activities (92±8 vs. 102±39 GBq/µmol) were obtained using 0.75 µmol and 1 µmol precursor, respectively, while the RCY slightly decreased using a lower amount of precursor (32±8 % vs. 40±3 %). Control experiments with a higher precursor amount (2 µmol) also did not result in higher RCY or molar activity (24±5%, 99±69 GBq/µmol).

Furthermore, the influence of some other parameters was investigated: type of base and complexant, reaction time and solvent (see Table S5 in SI). The parameters were chosen based on the optimization with difluoro­iodomethane.^13^ Variation of the type of base and complexant (at starting activities of 5 GBq) did not bring any improvement in RCY or molar activity: whereas KHCO_3_/K_222_ behaved similarly to K_2_CO_3_/K_222_ (RCY 57±2 % and A_m_ 27±5 GBq/µmol vs. RCY 55±4 % and A_m_ 25±7 GBq/µmol, respectively), K_2_CO_3_/18-cr-6 led to a drop in RCY (26±12 %) and a highly variable molar activity (42±40 GBq/ µmol). The reaction time did not have an influence on the RCY or molar activity; similar results were obtained after 1 and 10 min reaction with 5 GBq starting activity (RCY 58±2 % and A_m_ 32±21 GBq/µmol vs. RCY 55±4 % and A_m_ 25±7 GBq/µmol, respectively). As alternative solvents, THF and DMF were tested in a reaction with 5 GBq starting activity. With THF, lower RCYs (38±6 %) but slightly higher molar activities (43±8 GBq/µmol) were found compared to MeCN. In DMF the reaction did not proceed very well, RCYs were too low (10±5 %) for molar activity determination. Based on these results it was decided to keep using K_2_CO_3_/K_222_ in MeCN and a reaction time of 10 min.

Finally, the performance of the triazolium precursor **4** in the [^18^F]fluoroform synthesis was also evaluated using the Neptis® perform synthesis module. The same setup and synthesis sequence was used as we described earlier for difluoroiodomethane, and benzophenone was used as a model substrate for molar activity determination.^13^ In initial experiments, we observed molar activities that surpassed our quantification limit (A_m_ > 300 GBq/µmol, n = 3). However, RCYs were highly variable and mostly very low (RCY = 10±9 % over 3 experiments), and the high molar activity results could not be repeated. After slightly adjusting the automated procedure to increase the yield and get more reliable results, we were able to obtain [^18^F]fluoroform with an overall yield of 14±2 % and a molar activity of 153±14 GBq/µmol (n = 3). The heating time of the reactor before precursor addition was crucial and should be at least 2 to 2.5 minutes to guarantee the reaction temperature to be 80 °C. Compared to the results previously obtained with difluoroiodomethane (RCY = 9±2 %, A_m_ = 87±13 GBq/µmol),^13^ the triazolium precursor was superior in the automated synthesis since the molar activity was almost twice as high while the RCY was comparable to slightly better as well.

## Considerations regarding the reaction mechanism

In the above-mentioned investigation, triazolium precursor **4** provided good RCYs and molar activities in the manual (RCY = 40±3 %, A_m_ = 102±39 GBq/µmol, Table 2) as well as in the automated synthesis (RCY = 14±2 %, A_m_ =153±14 GBq/µmol). We hypothesize that the high molar activities obtained with precursor **4** can be explained by the reaction mechanism of the precursor reacting with [^18^F]fluoride and base.

In literature, it was proposed that the reaction of the triazolium precursor **4** with [^19^F]fluoride proceeds via three competing routes (a), (b), and (c): route (a) as the fluoroform formation via S*_N_*2 reaction, route (c) as the fluoroform formation via difluorocarbene, and route (b) as demethylation of the precursor (Scheme 3).^15^ Additionally, under radiochemistry conditions there could be a route (d) which is induced by the base present in the reaction and also leads to fluoroform formation via the difluorocarbene.

Our hypothesis was that the reaction mechanism of triazolium precursor **4** with [^19^F]fluoride could be reflected in the radiochemistry results: If the reaction of triazolium precursor **4** with [^18^F]fluoride under the basic radiofluorination conditions would proceed predominantly via routes (c) and (d) only [^18^F]fluoroform **11** with low molar activity would be obtained due to formation of difluorocarbene **17** (Scheme 3). Difluorocarbene has previously been proposed to release [^19^F]fluoride competing with [^18^F]fluoride in the [^18^F]fluoroform formation and therefore leading to isotopic dilution of the product.^12^ Route (a) in contrast would be able to provide [^18^F]fluoroform **11** with very high molar activity since no [^19^F]fluoride is released via this route.

Our radiochemistry data support this hypothesis and indicates that there is an equilibrium between routes (a), (c) and (d) for the [^18^F]fluoroform formation, which can be pushed towards one route or the other by controlling the amount of base present. High amounts of base result in [^18^F]fluoroform with low molar activity, indicating that route (d) is predominant in this case. Low amounts of base result in less route (d) and result in high molar activity, suggesting that the predominant pathway here is route (a).

To support our radiochemistry findings regarding the reaction mechanism we determined the reaction order of the reaction of triazolium precursor **4** with [^19^F]fluoride to fluoroform **16** in a time-dependent ^1^H NMR study (Table 5). Precursor **4** was reacted with 2 equivalents of CsF in CD_3_CN at 80 °C and yields of **4** and **10** were determined by ^1^H NMR. In the case of a first order kinetic behaviour, the plot of ln[RX] versus *t* will be linear (ln[RX] = -*kt* + ln[RX]_0_ ([RX] = concentration of **4**)). In the case of a second order behaviour, the plot of 1/[RX]$\frac{1}{[RX]}$ versus *t* will be linear (1/[RX] = 1/[RX]_0_ + *k’t*). The two graphs from the experimental results showing ln[RX] versus *t* and 1/[RX] versus *t* are displayed in Figures 4a and 4b, respectively. When adding linear trendlines to both graphs *r*^2^ in graph 4b is higher than that in graph 4a (*r* = correlation coefficient). Thus, the plot of 1/[RX] versus *t* is closest to linearity. Next, theoretical half-lives (*t*_1/2_) were calculated using the rate constants *k* and *k’* obtained from the trendlines of graphs 4a and 4b. In the case of the first order behaviour, *t*_1/2_ will be 2.057 h (*t*_1/2_ = ln2/*k*). In the case of the second order behaviour, *t*_1/2_ will be 1/(10.009[RX]_0_) (*t*_1/2_= 1/*k’*[RX]_0_). As the number of *t*_1/2_ elapsed increases to 1, 2, 3, the concentration of **4** decreases to 1/2, 1/4, 1/8. The theoretical plots of [RX] versus *t* of first order and second order are shown in Figure 4c. When comparing the experimental values with theoretical values, experimental values are more similar to values of theoretical second order_._ Overall, we propose that the kinetics of the reaction is second order, which means that the reaction proceeds via a S*_N_*2 process, route (a), under these reaction conditions.

# Conclusions

The triazolium precursor **4** is a valuable alternative to the already known precursors **1**-**3** for the [^18^F]fluoroform synthesis due to several reasons: 1) the triazolium precursor is a solid and therefore easier to handle than the gaseous difluoroiodomethane, 2) most of the previous findings with difluoroiodomethane concerning the [^18^F]fluoroform formation reaction also apply to the triazolium precursor and can therefore be adopted, 3) the triazolium precursor provides one of the highest molar activities of [^18^F]fluoroform observed so far, especially in the automated synthesis on the Neptis® perform module (A_m_ = 153±14 GBq/µmol, dc, EOS). However, careful control of the reaction conditions is crucial, since the optimal range of base amount and temperature is very narrow. Furthermore, optimal conditions need to be adjusted for different amounts of [^18^F]fluoride used in the reaction. This represents a major difference to difluoroiodomethane, that generally tolerates a broader range of reaction conditions. Nonetheless, triazolium precursor **4** is a valuable addition to the ^18^F-trifluoromethylation chemistry toolbox and might be of particular value in the synthesis of PET tracers that require high molar activities.

# Acknowledgements

The project is financially supported by the Dutch Research Council (NWO) grant no. 731.015.413 and BV Cyclotron VU (beneficiary: D. Vugts) and by The Radiation Technology R&D program through the National Research Foundation of Korea funded by the Ministry of Science, ICT & Future Planning (2016M2A2A7A03913537) (beneficiary: D. Y. Chi).

# Conflict of interests

Given her role as editor of this journal, Danielle J. Vugts had no involvement in the peer-review of this article. Full responsibility for the peer-review process for this article was delegated to Prof. Michael Kassiou.

# Data availability statement

The data that support the findings of this study are available from the corresponding author upon reasonable request.

# References

1. Gillis EP, Eastman KJ, Hill MD, Donnelly DJ, Meanwell NA. Applications of Fluorine in Medicinal Chemistry. *J Med Chem*. 2015;58(21):8315-8359. doi:10.1021/acs.jmedchem.5b00258

2. Meanwell NA. Fluorine and Fluorinated Motifs in the Design and Application of Bioisosteres for Drug Design. *J Med Chem*. 2018;61(14):5822-5880. doi:10.1021/acs.jmedchem.7b01788

3. Wang J, Sánchez-Roselló M, Aceña JL, et al. Fluorine in pharmaceutical industry: Fluorine-containing drugs introduced to the market in the last decade (2001-2011). *Chem Rev*. 2014;114(4):2432-2506. doi:10.1021/cr4002879

4. Riss PJ, Aigbirhio FI. A simple, rapid procedure for nucleophilic radiosynthesis of aliphatic [18F]trifluoromethyl groups. *Chem Commun*. 2011;47(43):11873-11875. doi:10.1039/c1cc15342k

5. Matthews PM, Rabiner EA, Passchier J, Gunn RN. Positron emission tomography molecular imaging for drug development. *Br J Clin Pharmacol*. 2012;73(2):175-186. doi:10.1111/j.1365-2125.2011.04085.x

6. Tredwell M, Gouverneur V. 18F Labeling of Arenes. *Angew Chemie Int Ed*. 2012;51(46):11426-11437. doi:10.1002/anie.201204687

7. Van Der Born D, Pees A, Poot AJ, Orru RVA, Windhorst AD, Vugts DJ. Fluorine-18 labelled building blocks for PET tracer synthesis. *Chem Soc Rev*. 2017;46(15):4709-4773. doi:10.1039/c6cs00492j

8. Huiban M, Tredwell M, Mizuta S, et al. A broadly applicable [18F]trifluoromethylation of aryl and heteroaryl iodides for PET imaging. *Nat Chem*. 2013;5(11):941-944. doi:10.1038/nchem.1756

9. van der Born D, Herscheid JDM, Orru RVA, Vugts DJ. Efficient synthesis of [18F]trifluoromethane and its application in the synthesis of PET tracers. *Chem Commun*. 2013;49(38):4018-4020. doi:10.1039/c3cc37833k

10. van der Born D, Sewing C, Herscheid JDM, Windhorst AD, Orru RVA, Vugts DJ. A universal procedure for the [18F]trifluoromethylation of aryl iodides and aryl boronic acids with highly improved specific activity. *Angew Chemie Int Ed*. 2014;53(41):11046-11050. doi:10.1002/anie.201406221

11. Rühl T, Rafique W, Lien VT, Riss PJ. Cu(I)-mediated 18F-trifluoromethylation of arenes: Rapid synthesis of 18F-labeled trifluoromethyl arenes. *Chem Commun*. 2014;50(45):6056-6059. doi:10.1039/c4cc01641f

12. Ivashkin P, Lemonnier G, Cousin J, et al. [18F]CuCF3 : a [18F]trifluoromethylating agent for arylboronic acids and aryl iodides. *Chem - A Eur J*. 2014;20(31):9514-9518. doi:10.1002/chem.201403630

13. Pees A, Windhorst AD, Vosjan MJWD, Tadino V, Vugts DJ. Synthesis of [18F]Fluoroform with High Molar Activity. *European J Org Chem*. 2020;2020(9):1177-1185. doi:10.1002/ejoc.202000056

14. Yang BY, Telu S, Haskali MB, Morse CL, Pike VW. A Gas Phase Route to [18F]fluoroform with Limited Molar Activity Dilution. *Sci Rep*. 2019;9(1):14835. doi:10.1038/s41598-019-50747-3

15. Chai JY, Cha H, Lee S-S, Oh Y-H, Lee S, Chi DY. Mechanistic study of nucleophilic fluorination for the synthesis of fluorine-18 labeled fluoroform with high molar activity from N-difluoromethyltriazolium triflate. *RSC Adv*. 2021;11:6099-6106. doi:10.1039/D0RA09827B

16. Chai JY, Cha H, Kim HB, Chi DY. Selective addition reactions of difluoromethyltriazoles to ketones and aldehydes without the formation of difluorocarbene. *Tetrahedron*. 2020;76(31-32):131370. doi:10.1016/j.tet.2020.131370

17. Pees A, Sewing C, Vosjan MJWD, et al. Fast and reliable generation of [18F]triflyl fluoride, a gaseous [18F]fluoride source. *Chem Commun*. 2018;54(72):10179-10182. doi:10.1039/c8cc03206h

# Appendix

## Tables

**Table 1** Investigation of stability of precursor **4** in presence of K_2_CO_3_ and K_222_*^a^*

| **entry** | **temp.** | **K_2_CO_3_**  **(equiv)** | **K_222_**  **(equiv)** | **yield (%)*^b^*** | |
| --- | --- | --- | --- | --- | --- |
|  |  |  |  | **4** | **10** |
| 1 | rt | 0.15 | 0.35 | 99.7 | 0.3 |
| 2 | rt | 1.5 | 3.5 | 40 | 60 |
| 3*^c^* | 40 °C | 0.15 | 0.35 | 95 | 5 |
| 4*^c^* | 80 °C | 0.15 | 0.35 | 91 | 9 |

*^a^*Reactions were carried out on a 0.5 µmol reaction scale of triazolium precursor **4** in 0.5 mL of CD_3_CN in a sealed NMR tube. n = 1. *^b^* ^1^H NMR yields. *^c^*After reaction time, the mixture was cooled to 0 °C.

**Table 2** Temperature dependency of the [^18^F]fluoroform synthesis with the triazolium precursor **4** using different [^18^F]fluoride amounts; 1 µmol prec., 0.15 µmol K_2_CO_3_, 0.35 µmol kryptofix 222, 10 min, 1 mL MeCN; dc.

| **entry** | **^18^F^-^**  **(GBq)** | **temp.**  **(°C)** | **RCY**  **(%)** | **A_m_**  **(GBq/µmol)** | **n** |
| --- | --- | --- | --- | --- | --- |
| 1 | 0.5 | 40 | 52±6 | n.d. | 3 |
| 2 | 0.5 | 80 | 28±13 | n.d. | 3 |
| 3 | 5 | 40 | 55±4 | 25±7 | 3 |
| 4 | 5 | 80 | 46±3 | 36±13 | 3 |
| 5 | 25 | 40 | 25±7 | 67±20 | 3 |
| 6 | 25 | 80 | 40±3 | 102±39 | 4 |

**Table 3** RCY and A_m_ of [^18^F]fluoroform synthesized with the triazolium precursor **4** starting from 5 GBq [^18^F]fluoride; 1 µmol prec., 10 min, 1 mL MeCN, 40 °C, dc, n=3, n.d.=not determined, ^a^ reaction conditions: 80 °C, *due to low yields A_m_ could only be determined once.

| **entry** |  |  | **triazolium precursor** | | **CHIF_2_ precursor (Lit. data**^13^**) *^a^*** | |
| --- | --- | --- | --- | --- | --- | --- |
|  | **K_2_CO_3_**  **(µmol)** | **K_222_**  **(µmol)** | **RCY**  **(%)** | **A_m_**  **(GBq/µmol)** | **RCY**  **(%)** | **A_m_**  **(GBq/µmol)** |
| 1 | 0.015 | 0.035 | 0±0 | n.d. | 18±3 | 36±30 |
| 2 | 0.038 | 0.088 | 11±12 | 314 (n = 1)* | 19±6 | 38±44 |
| 3 | 0.075 | 0.175 | 49±10 | 75±40 | 38±2 | 25±12 |
| 4 | 0.113 | 0.263 | 58±3 | 39±4 | n.d. | n.d. |
| 5 | 0.150 | 0.350 | 55±4 | 25±7 | 44±1 | 18±2 |
| 6 | 0.750 | 1.750 | 27±2 | 7±3 | 40±1 | 5±1 |
| 7 | 1.500 | 3.500 | 17±2 | 5±2 | 23±2 | 4±2 |

**Table 4** RCY and A_m_ of [^18^F]fluoroform synthesized with the triazolium precursor **4** using 25 GBq [^18^F]fluoride; 1 µmol prec., 10 min, 1 mL MeCN, 40 °C; dc.

| **entry** | **K_2_CO_3_**  **(µmol)** | **K_222_**  **(µmol)** | **RCY**  **(%)** | **A_m_**  **(GBq/µmol)** | **n** |
| --- | --- | --- | --- | --- | --- |
| 1 | 0.075 | 0.175 | 0±0 | n.d. | 2 |
| 2 | 0.150 | 0.350 | 25±7 | 67±20 | 3 |
| 3 | 0.300 | 0.700 | 38±1 | 57±4 | 2 |
| 4 | 0.450 | 1.050 | 35±1 | 30±1 | 2 |

**Table 5** Determining the reaction order. Reaction was carried out on a 0.084 mmol reaction scale of triazolium precursor **4** in 0.75 mL of CD_3_CN in a sealed NMR tube; * ^1^H NMR yields.

| **entry** | **time (h)** | **yield (%)*^*^*** | | **[RX]**  **(M)** | **ln[RX]** | **1/[RX] (1/M)** |
| --- | --- | --- | --- | --- | --- | --- |
|  |  | **4** | **10** |  |  |  |
| 1 | 0 | 100 | 0 | 0.11200 | -2.189 | 8.929 |
| 2 | 0.167 | 82 | 18 | 0.09184 | -2.388 | 10.889 |
| 3 | 0.333 | 74 | 26 | 0.08288 | -2.490 | 12.066 |
| 4 | 0.5 | 67 | 33 | 0.07504 | -2.590 | 13.326 |
| 5 | 2 | 34 | 66 | 0.03808 | -3.268 | 26.261 |
| 6 | 3 | 24 | 76 | 0.02688 | -3.616 | 37.202 |
| 7 | 5 | 15 | 85 | 0.01680 | -4.086 | 59.524 |
| 8 | 6 | 13 | 87 | 0.01456 | -4.229 | 68.681 |

## Figures and Schemes

**Scheme 1** Synthesis of triazolium salt **4**.

**Scheme 2** Generation of reactive [^18^F]fluoride via gaseous [^18^F]triflyl fluoride **13,** followed by [^18^F]fluoroform **11** synthesis with the triazolium precursor **4** and subsequent model reaction to the UV active carbinol **15** for molar activity determination.

**Scheme 3** Proposed reaction mechanism for the reaction of triazolium precursor **4** with [^18^F]fluoride.

**Figure 1** Different precursors for [^18^F]trifluoromethylation: methyl chlorodiﬂuoroacetate **1**, difluoroiodomethane **2**, difluoromethylsulfonium salt **3**, and triazolium salt **4**.

**Figure 2** Temperature dependency of the [^18^F]fluoroform synthesis with the triazolium precursor **4**; 500 MBq dry [^18^F]fluoride, 1 µmol prec., 0.15 µmol K_2_CO_3_, 0.35 µmol kryptofix 222, 10 min, 1 mL MeCN; dc, n = 3.

**Figure 3** Dependency of the [^18^F]fluoroform synthesis on the triazolium precursor amount; 500 MBq dry [^18^F]fluoride, triazolium precursor **4**, 0.15 µmol K_2_CO_3_, 0.35 µmol kryptofix 222, 10 min, 40 °C, 1 mL MeCN; dc, n = 3 (n = 6 for 1 and 5 µmol).


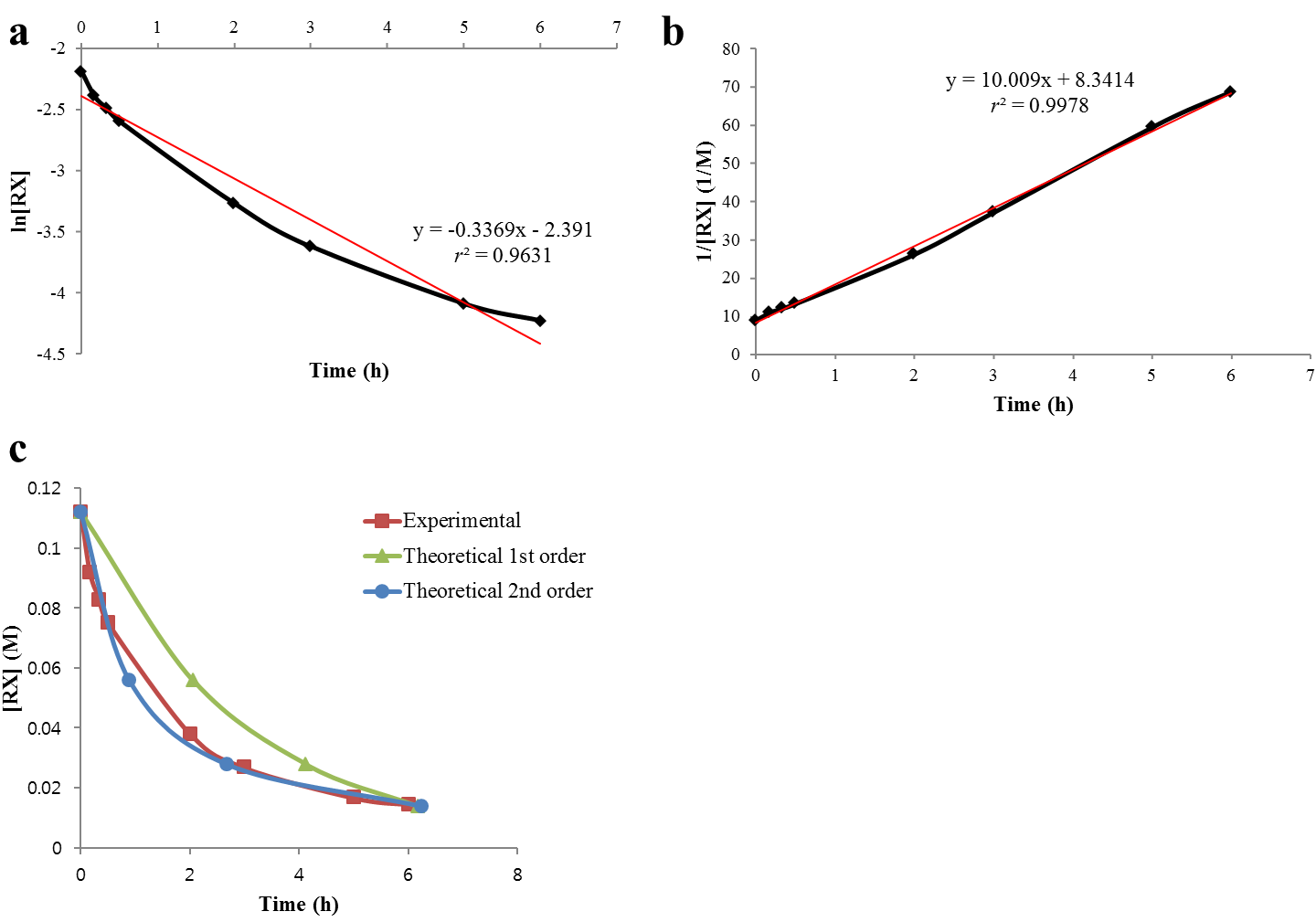


**Figure 4** Plots of (a) ln[RX] versus t, (b) 1/[RX] versus t, and (c) [RX] versus t.
